# Dynamic Modeling and Analysis of the Cross-Talk between Insulin/AKT and MAPK/ERK Signaling Pathways

**DOI:** 10.1371/journal.pone.0149684

**Published:** 2016-03-01

**Authors:** Yaman Arkun

**Affiliations:** Department of Chemical and Biological Engineering, Koc University, Rumeli Feneri Yolu, 34450 Sariyer, Istanbul, Turkey; Thomas Jefferson University, UNITED STATES

## Abstract

Feedback loops play a key role in the regulation of the complex interactions in signal transduction networks. By studying the network of interactions among the biomolecules present in signaling pathways at the systems level, it is possible to understand how the biological functions are regulated and how the diseases emerge from their deregulations. This paper identifies the key feedback loops involved in the cross-talk among the insulin-AKT and MAPK/ERK signaling pathways. We developed a mathematical model that can be used to study the steady-state and dynamic behavior of the interactions among these two important signaling pathways. Modeling analysis and simulation case studies identify the key interaction parameters and the feedback loops that determine the normal and disease phenotypes.

## Introduction

**Fig 1:**
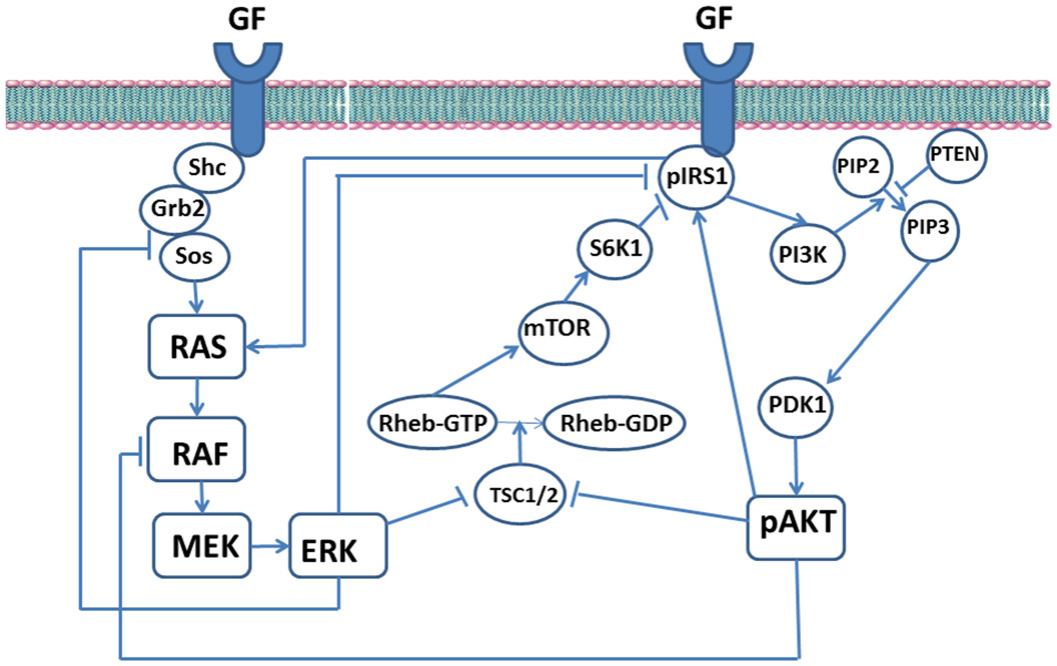
Pathway Structure of Insulin-MAPK/ERK Interactions.

In this study we model the major feedback loops that regulate the cross-talk between insulin-AKT and MAPK/ERK signaling pathways shown in Fig 1. Insulin-AKT signaling pathway is responsible for the regulation of glucose in the blood. Type-2 diabetes is characterized by ineffective use of insulin, called insulin resistance [1]. AKT is the key protein kinase involved in the metabolic actions of insulin [2]. AKT is activated through a PI3K/PTEN dependent mechanism and promotes glucose uptake by translocating GLUT-4 to the cell surface [2–7]. Activated AKT (pAKT) drives cell proliferation [5] and also enhances vasodilation by stimulating NO production [8,9]. Defects in insulin mediated activation of AKT can lead to insulin resistance and promote Type-2 diabetes.

MAPK (Mitogen Activated Proten Kinase) pathway consists of a three-level Ras/Raf/MEK/ERK signaling cascade which is initiated by the binding of epidermal growth factor (EGF) to its receptor. In particular, after binding of EGF, Shc/Grb2/SOS complex forms. Ras, which is a small GTP binding protein, interacts with SOS and it gets converted to its active conformation by exchanging GDP for GTP. Active Ras can then recruit Raf to the cell membrane and start the sequential phosphorylation of the Raf/MEK/ERK signaling cascade.

Activated ERK (pERK) phosphorylates SOS and disassembles the Grb2-SOS complex and deactivates Ras [10–12]. This feedback inhibition can explain the different responses of the MAPK pathway in EGF and NGF signaling [13].

In response to different growth factors, MAPK cascade of signaling proteins controls diverse cellular functions such as growth, differentiation, apoptosis and proliferation. The type of biological response exerted by MAPK pathway depends on the cell type, the amplitude and duration of the external stimulus [12, 14, 15]. In many cancer and drug resistance cases, MAPK/ERK pathway has been found to be mutated or overexpressed [16, 17]. Deregulation of ERK signaling is linked to tumorigenesis [18]. MAPK and AKT signaling systems are known to interact through different paths. For example overexpressed ERK impairs insulin signaling and induces insulin resistance [19]. At high doses of insulin growth factor, active AKT phosphorylates RAF at serine residue *Ser*^259^ and thus suppresses the activity of RAF-MEK-ERK signaling pathway [14,20]. Therefore, these pathways have been a common target for cancer therapy and treatment of diabetes [16, 21, 22].

In our modeling approach, we first construct the signaling pathways and the regulatory feedback loops using literature knowledge. Next, mass-action kinetics and conservation laws are used to model the chemical reaction network of the signaling biomolecules. The model is in the form of nonlinear differential equations which can be used to predict the dominant steady-state and dynamic signaling interactions. Using this model we postulate possible new mechanisms provided by the feedback loops to explain cellular responses.

## Methods

AKT and MAPK signaling pathways and their interactions are illustrated in Fig 1. Nodes represent the molecules and directed edges among them represent the molecular interactions. Pointed arrows stand for activations; blunt arrows denote the inhibitory effects.

**Fig 2:**
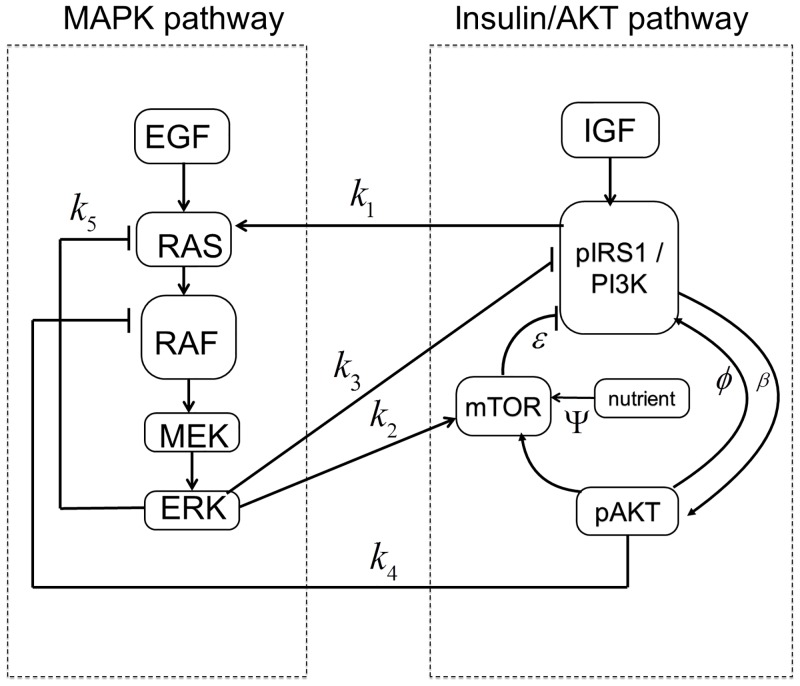
Simplified signaling network.

In order to reduce the complexity of the cellular network and facilitate the subsequent modeling and analysis, Fig 1 is simplified to Fig 2 by lumping some of the intermediate molecular interactions. In Fig 2 the edges crossing the boundaries of the individual subsystems (i.e. insulin/AKT and MAPK/ERK) represent the signals that are involved in the cross-talk among these subsystems. Each of the cross-talk edges is labeled by a variable representing the gain or strength of the interaction as summarized in Table 1. The physical origin of each cross-talk is explained next.

**Table 1:** Interactions appearing in the cross-talk in Fig 2.

Interaction parameters	Type of Interaction
*k*_1_	Insulin dependent activation of ERK
*k*_2_	ERK inhibits pIRS1 by activating mTOR
*k*_3_	ERK inhibits pAKT through Gab1-PI3K
*k*_4_	pAKT inhibits ERK

*Insulin dependent activation of ERK*: Growth factor insulin binds to its receptor IR and promotes the tyrosine phosphorylation of its substrate IRS1. Tyrosine phosphorylated IRS1 (pIRS1) stimulates the MAPK signaling cascade by catalyzing Shc which interacts with the Grb2-SOS complex. This is followed by the stepwise activation of Ras, Raf, MEK, and extracellular signal regulated kinase (ERK) [10, 23].

*ERK inhibits pIRS1 by activating mTOR*: Activated ERK phosphorylates a distinct site on TSC2 leading to a greater activation of mTOR [24,25]. mTOR activates S6K which phosphorylates and inhibits IRS1 [24–27]. It is reported that inhibition of mTOR by the anticancer drug ramapycin leads to MAPK activation [28]. This is attributed to the mechanism in which inhibition of mTOR upregulates IRS-1 and increases Ras downstream of IRS-1.

*Activated ERK inhibits pAKT*: The docking protein GAB1 plays an important role in the control of MAPK and PI3K/Akt signaling pathways. GAB1 extends the duration of MAPK signaling by stimulating PI3K/Akt activation [10,11]. Epithermal Growth Factor (EGF)-stimulated ERK activation decreases tyrosine phosphorylation of Grb2-associated binder Gab1 and down-regulates the association of Gab1 with PI3K. This decreases the activation of AKT downstream [10]. ERK has been found to be activated in insulin resistant cases and inhibition of glucose uptake was completely reversed by ERK1/2 inhibitor PD98059 inhibitor [29].

*Phosphorylaed AKT (pAKT) inhibits ERK*: At high doses of insulin growth factor, active AKT *(pAKT*) phosphorylates RAF at serine residue *Ser*^259^ and thus inhibits activation of the RAF-MEK-ERK signaling pathway [14,20]. In human breast cancer cell lines, this cross-talk between AKT and RAF-MEK-ERK pathways shifts the cellular response from cell cycle arrest to proliferation [14,20].

### Feedback Loops

Cellular networks are complex systems made up of multiple pathways that interact in a non-simple way. Signaling pathways collectively maintain their normal biological operating conditions through cooperation of coupled negative and positive feedback loops [30–32]. Loss of feedback loops or the coordination among them results in abnormal disease state. Upon closer inspection of the network given in Fig 2, one can identify several feedback loops. A feedback loop exits, if starting from a node, one can return to that node by following the edges in the direction of their arrows. The sign of these feedback loops is either positive or negative as explained next.

#### 1. The feedback loops within the insulin/AKT signaling pathway

*pIRS→pAKT→pIRS1 positive feedback loop*: AKT is activated by a PI3K dependent mechanism [2–7]. In order to maintain the tyrosine phosphorylation of IRS1, activated AKT (or pAKT) activates pIRS1 and forms a positive feedback loop [33–34].

*pIRS1→pAKT→pIRS1 negative feedback loop*: pAKT inhibits pIRS1 through activation of mTOR and S6K [24, 35, 36] and this constitutes a negative feedback loop. pAKT should be able to switch between high and low values according to the cellular and extracellular conditions since the translocation of GLUT4 to the plasma membrane and glucose uptake is an all or none type process [37]. Combination of the positive and negative loops ensures the bistable switching response which is necessary for the insulin sensitivity.

#### 2. The feedback loop within the MAPK signaling pathway

*RAS→RAF→MEK→ERK→RAS negative feedback loop*: ERK promotes the phosphorylation of SOS and disassociates the Grb2-SOS complex which terminates RAS activation [12, 13, 38]. This feedback inhibition plays an important role in determining the duration of the MAPK cascade’s activation [13, 39].

#### 3. Inter-pathway feedback loops

In addition to the above internal feedback loops of the individual pathways, there also exist the following feedback loops across the pathways due to the cross-talk. (see Fig 2). These loops are:

*pIRS1→ERK→mTOR→pIRS1 Negative feedback loop*: Insulin dependent activation of ERK is followed by ERK’s inhibition of the AKT pathway through mTOR.

*pIRS1→pAKT→ERK→mTOR→pIRS1 Positive feedback loop*: Two inhibitions in a loop form a positive feedback. Accordingly, inhibitory action of pAKT on ERK may be beneficial since it can repair pIRS1 which is inhibited by ERK.

*ERK→pIRS1/PI3K→ERK Negative feedback loop*: ERK can inhibit the insulin stimulated IRS-1/PI3K association by down-regulating Gab1’s association with PI3K. This inhibition together with the activation of ERK by pIRS1 forms a negative feedback loop.

*pIRS1→pAKT→ERK→pIRS1 Positive feedback loop*: Similar to the other positive feedback loop, two different inhibitions in a sequence form a positive loop.

### Development of the Dynamic Model

In [34] Wang developed a mathematical model for the AKT signaling pathway to investigate system-level mechanisms of cell growth and metabolism. In [40] we reduced this original AKT model to a two-state reduced-order model which is easier to manipulate and extend to include interactions with multiple signaling pathways. Modeling of the MAPK/ERK signaling pathway has been extensively studied in the literature. Huang and Ferrell [41] developed the first model for the MAPK cascade which consisted of differential and algebraic equations representing mass action kinetics for 22 species and 10 reactions. Since the original Huang and Ferrell model, many new MAPK models have been developed as reviewed in [42].

In this paper we combine the AKT model and the Huang-Ferrell’s MAPK model by including the dynamics of the cross-talk. The new model consists of 17 differential equations derived from the conservation law and mass action kinetics for the species shown in Fig 2 (see the S1 Text).

## Results and Discussion

### Analysis of individual pathways without the cross—talk

We first analyze each signaling subsystem (i.e. AKT and MAPK) separately by ignoring the cross-talk. This is next followed by the analysis of the two subsystems in the presence of the cross-talk. This allows us to draw conclusions on the effect of interactions between the two signaling subsystems. We are particularly interested in how the bistability property and response characteristics of the individual signaling pathways are affected by the intra-pathway feedback loops which govern the dynamics of the cross-talk.

The true values for most of the model parameters usually do not exist due to lack of reliable data. Therefore, we have chosen a nominal set of literature values which give typical bistable responses observed for the insulin-AKT and MAPK pathways. Specifically, the parameter values for the MAPK pathway and the AKT pathways are taken from [32] and [34], respectively. This is called the base case. The values and the units are given in Table A in S1 Text and Table B in S1 Text, respectively. The stimulus for the AKT pathway is the insulin level *γ* and it is normalized in terms of the model parameters as $\lambda =\;\frac{\gamma \beta k_{1}}{\delta \;k_{2}E_{2T}}$(see S1 Text). *E*1_*tot*_ is the total concentration of enzyme *E*1 which initiates the response of the MAPK cascade (see S1 Text). In the results to follow, AKT and ERK responses were calculated and plotted as a function of the inputs, *λ* and *E*1_*tot*_, by using MATCONT bifurcation toolbox [43].

It is well-established that the normal phenotype for the insulin-AKT signaling pathway exists when the cellular dynamic response to insulin is able to switch between two stable steady-states separated by an unstable steady-state [34]. Similarly insulin-stimulated GLUT4 translocation for glucose transport exibits bistable switch-like response to the insulin input. In cellular processes, bistability is a result of the presence of a positive feedback loop or two negative feedback loops which collectively create a positive feedback loop action [44]. This bistable behavior is usually a robust property meaning it is maintained for a wide range of parameter values [30, 34, 45]. In fact only large perturbations are expected to lead to the loss of bistability and the emergence of the disease states like diabetes, hypertension and cancer. In [34] Wang has mapped these phenotypes to the space of modeling parameters and has elucidated mechanisms for disease prevention and therapy. It was shown that the existence of normal phenotype or bistable switch-like response is determined by the relative strengths of positive and negative feedback loops indicated by the parameter *θ* = (Φ−εΨ) (see Fig 2). Normal operation of the insulin signaling pathway requires the positive feedback to be greater than the negative feedback i.e. *θ* > 0. If the negative feedback dominates persistently, bistability is lost and insulin sensitivity and type-2 diabetes develops.

**Fig 3:**
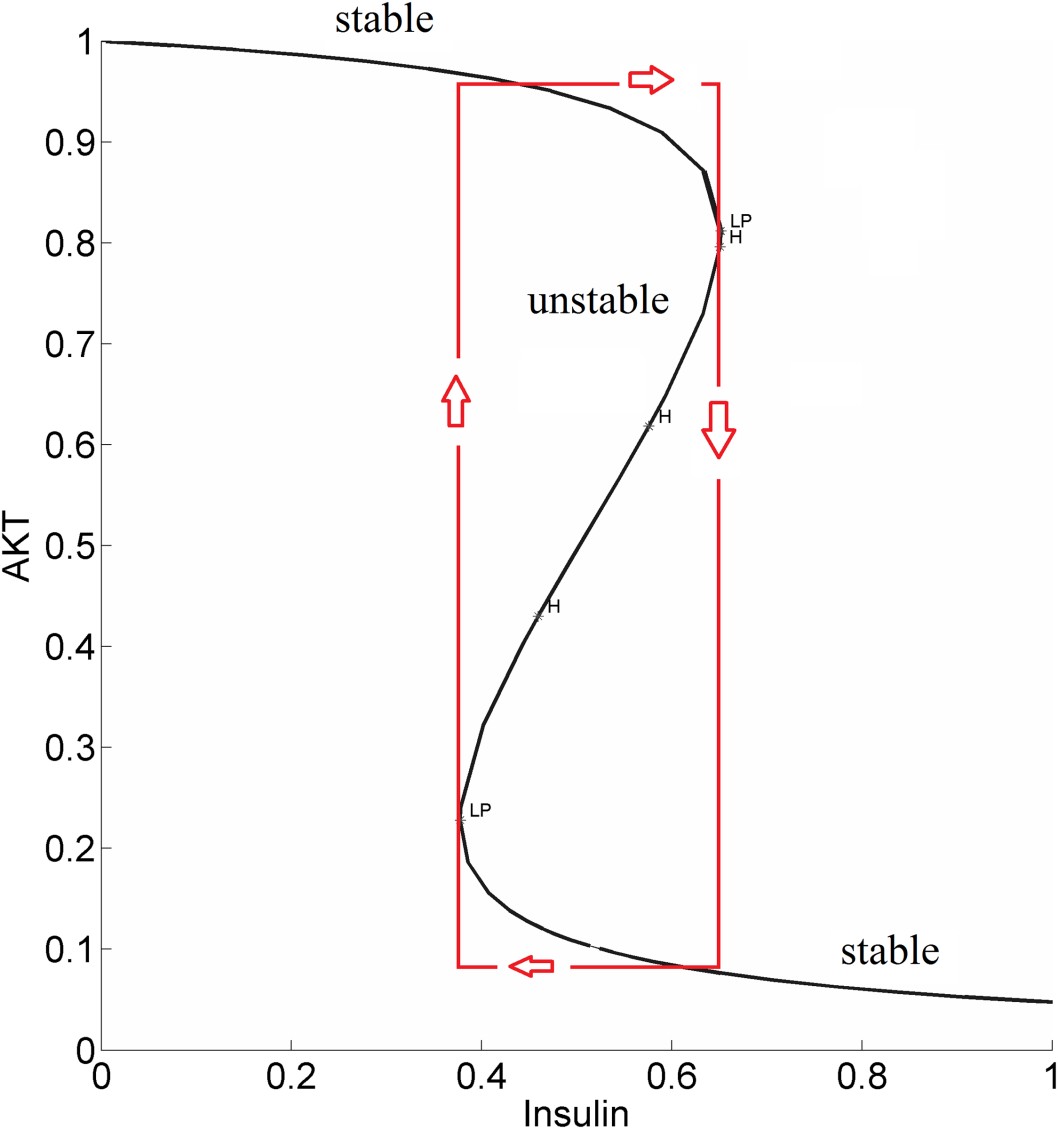
Steady-state response curve of AKT to insulin (parameter *λ* in the model) without the cross-talk. LP: Limit Point bifurcation also called the turning point. The switch between the low and high stable branches occurs at the turning points and it is shown by the arrows. H: Hopf bifurcation.

Fig 3 shows the sigmoidal bistable AKT response calculated from our model.

Due to high insulin sensitivity established by the positive and negative feedback actions, AKT is able to switch between its high (upper steady-state branch) and low (lower steady-state branch). When AKT is high, the cell has low nutrient level and requires glucose uptake. The system is at the upper steady-state. By an increase in insulin, AKT gets activated to pAKT and glucose is taken into the cell. The system switches to the lower steady-state. Withdrawing insulin enables the switch back to high AKT or low pAKT levels.

MAPK model developed by Huang and Ferrell predicts ultrasensitivity (bistable switch-like response curve) which increases down the MAPK cascade and it is robustly maintained for a wide range of concentrations and parameter values. This was also experimentally confirmed by the observations that ERK activation was switch-like in individual Xenopus oocytes [46]. Ultrasensitive switch-like responses of ERK are involved in the control of the cell fate [46, 47]. Later it was shown that bistability can arise from phosphorylation–dephosphorylation cycle at a single level of the signaling cascade without requiring any external feedback loop [48]. Qiao et al. [32] by using random parameter search and continuation algorithms showed that MAPK exhibits oscillatory and bistable responses for a significant range of parameter values.

**Fig 4:**
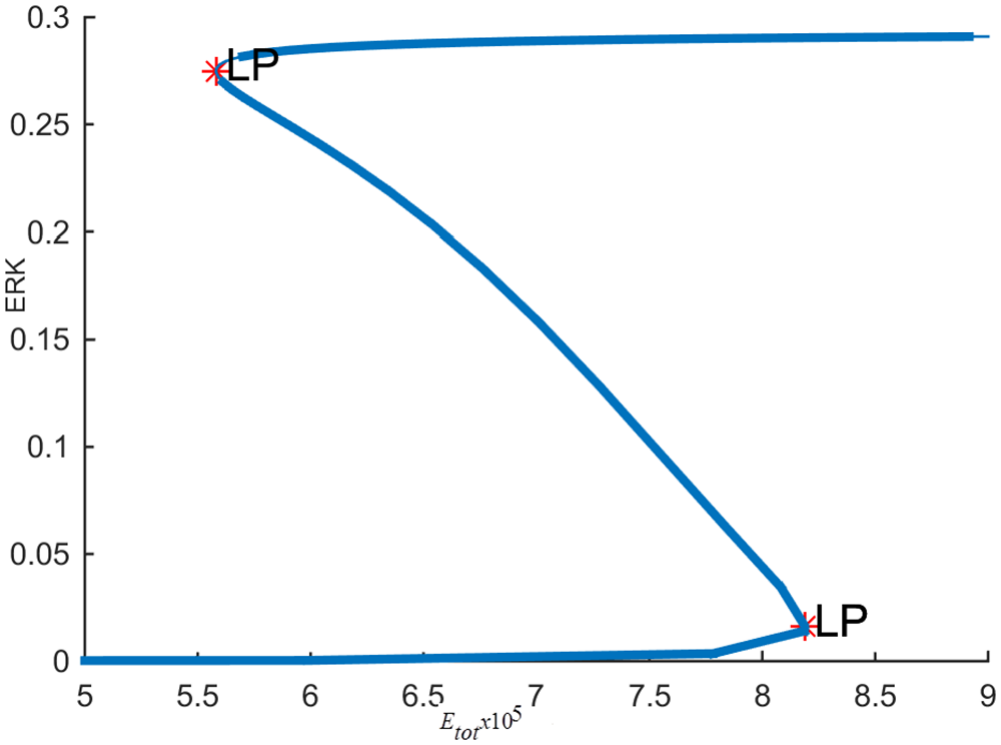
Steady-state response of ERK to *E*1_*tot*_ without the cross-talk.

**Fig 5:**
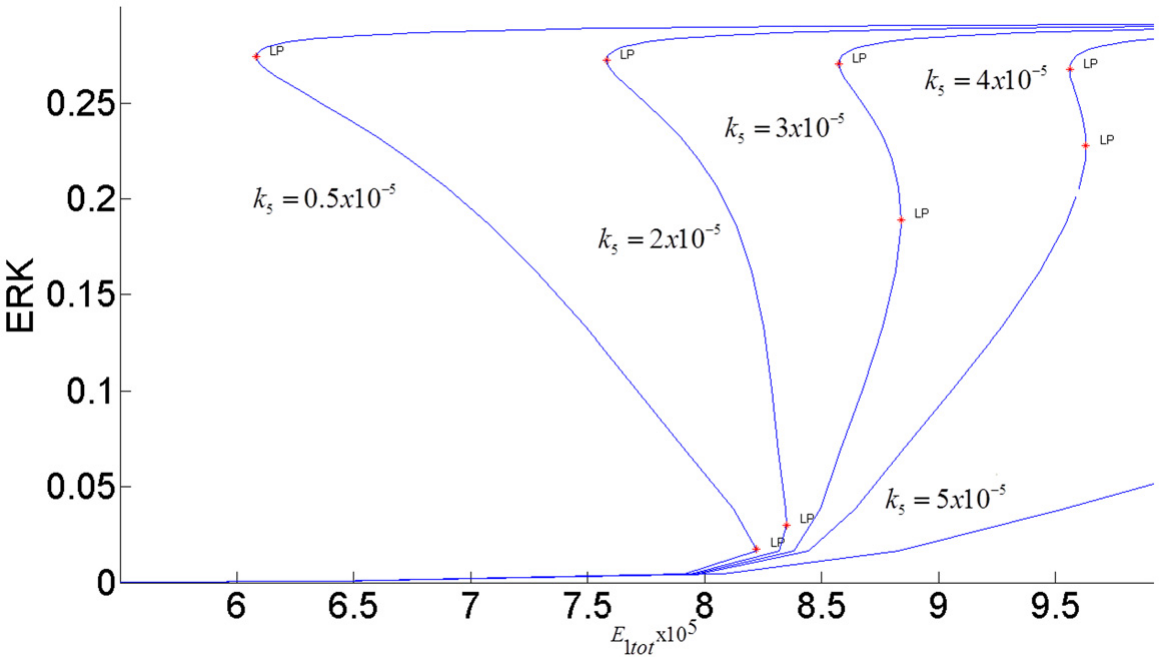
Effect of the feedback inhibition of RAS on ERK activation. Parameter *k*_5_ indicates the strength of the inhibitory feedback signal.

The steady-state response curve of ERK as a function of the stimulus *E*1_*tot*_ is shown in Fig 4. It is seen that model predicts the ultrasensitive switch-like response since the response curve is bistable. The effect of negative feedback from ERK to RAS is shown in Fig 5. As the gain of negative feedback *k*_5_ increases, ERK is inhibited more; therefore, the response curve shifts to the right and requires higher stimulus *E*1_*tot*_to be able to switch between its inactive and active steady-states. As feedback inhibition increases, the range of stimulus that sustains ERK activity gets smaller and eventually, when the feedback inhibition is high enough, ERK has a graded monostable response that changes only incrementally as the stimulus *E*1_*tot*_ increases.

#### The switching response of ERK signal can be modulated by the internal feedback and the growth factor stimulus to perform different biological functions

It is known that ERK responses to external stimulus exhibit different amplitudes and frequencies. In particular, the duration of ERK activity is a critical factor in determining its biological function. In addition, sustained ERK activation is necessary for inducing cyclin-D1 and G1 phase cell cycle progression [49]. It is also known that transient short term ERK signal induces *p*21^chip1^ which inhibits proliferation [15]. The frequency at which ERK switches between “on” and “off” sates determines how information is transmitted by the MAPK pathway [50]. As a possible mechanism, our model attributes the changes in the magnitude and duration of ERK signaling specifically to the modulation of the bistability of the MAPK pathway by two control agents: the internal negative feedback gain *k*_5_ and the external stimulus *E*1_*tot*_.

**Fig 6:**
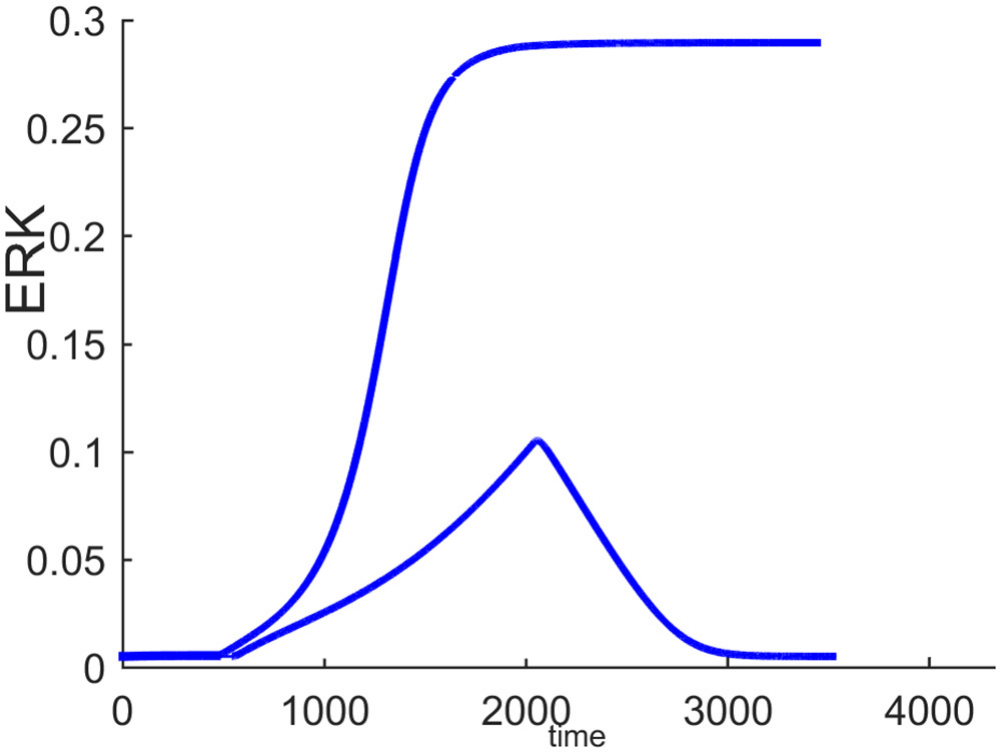
Dynamic responses of ERK to a pulse *E*1_*tot*_ stimulus with different negative feedback gains.

Fig 6 shows the switch-like dynamic response of ERK to a pulse stimulus. At time = 500, *E*1_*tot*_ is increased from its nominal value 8x10^-5^ to 8.5x10^-5^ and is held at this value for 1500 time units and then decreased back to its initial value of 8x10^-5^. With lesser negative feedback inhibition, ERK activity is persistently sustained at its high activation state.

*E*1_*tot*_ has to be decreased further below 6.08x10^-5^ for the ERK signal to switch back to its lower steady-state. When feedback inhibition increases, ERK activation cannot be sustained and a transient short term response is obtained. Thus, the model can generate different ERK responses to perform different biological functions by manipulating the strength of the internal feedback and the input stimulus. In [13] this same feedback regulation is shown to be an important factor in determining the differences between the responses of the MAPK cascade to different growth factors, NGF and EGF in particular.

### Analysis of the inter-pathway feedback loops

**Fig 7:**
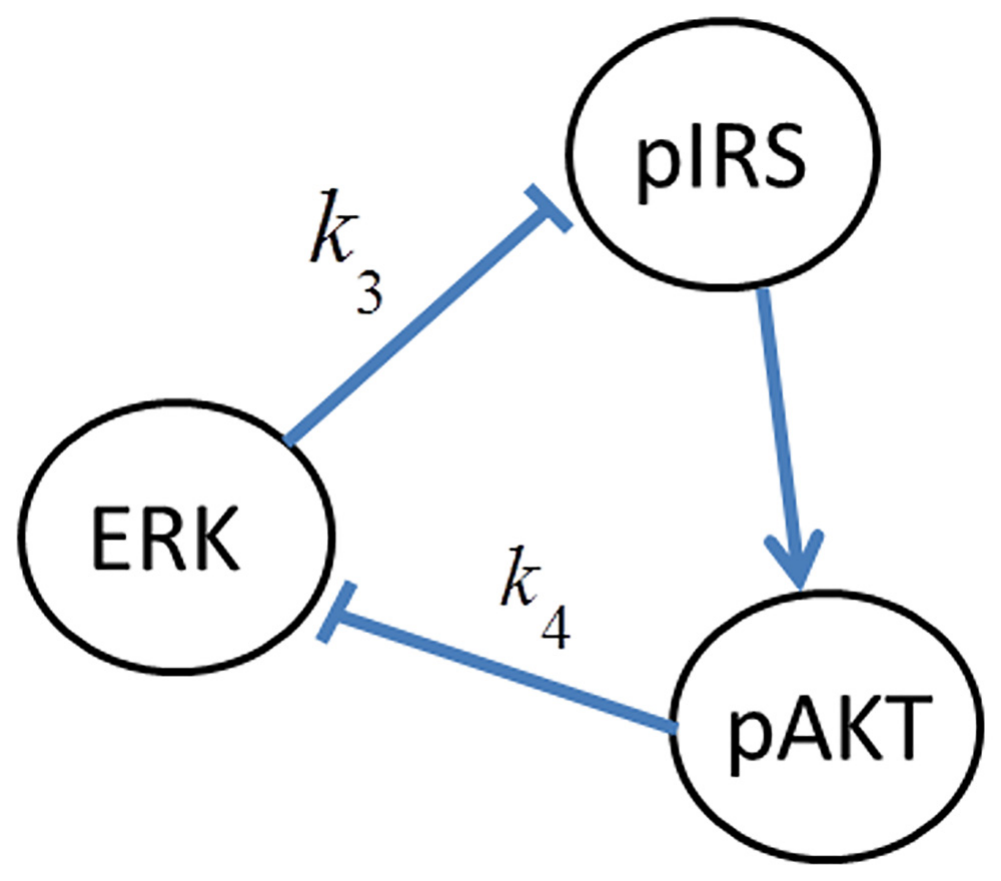
The positive feedback loop *FB*(*k*_3_,*k*_4_).

We next consider the effect of cross-talk by analyzing each inter-pathway feedback and the interactions listed in Table 1. We first study *pIRS1→pAKT→pERK→pIRS1* positive feedback loop shown in Fig 7.

The feedback loop in Fig 7 is represented by the parameters of the inter-pathway interactions that make up the loop i.e. as *FB*(*k*_3_,*k*_4_). All other cross-talk parameters are set to zero. In order to first analyze the open-loop effect of *k*_3_ or ERK’s inhibition of pIRS1, *FB*(*k*_3_,*k*_4_) is temporarily opened by setting *k*_4_ to zero.

*ERK inhibits pIRS1*: *k*_3_≠0 *and k*_4_ = 0.

**Fig 8:**
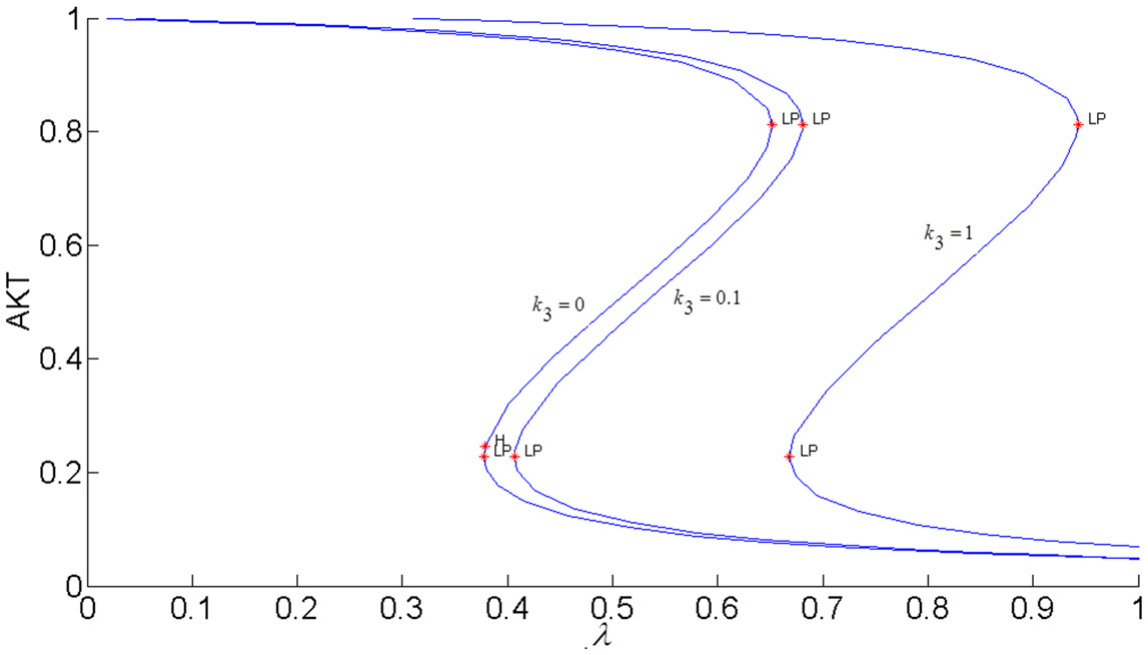
Steady state AKT responses when ERK inhibits pIRS1.*E*1_*tot*_ *=* 9×10^−5^. *k*_*4*_ *= 0*. Parameter *k*_3_ indicates the strength of the inhibition.

Epithermal Growth Factor (EGF)-stimulated ERK activation down-regulates the association of Gab1 with PI3K and the activation of AKT downstream i.e. pAKT decreases. This inhibition is represented by the parameter *k*_3_. Fig 8 shows how ERK inhibits the response of AKT. As the strength of ERK’s inhibition increases, the responses shift to the right and more insulin is needed to activate AKT. When *k*_3_ = 0.1, AKT is able to switch at the insulin levels of 0.41 and 0.67 at the LP points. But, when *k*_3_ = 1, AKT persists to stay at its high state for these insulin values and more insulin is required to move AKT to its low state and restore the switch-like behavior.

*pAKT inhibits ERK*: *k*_4_≠0 *and k*_3_ = 0.

**Fig 9:**
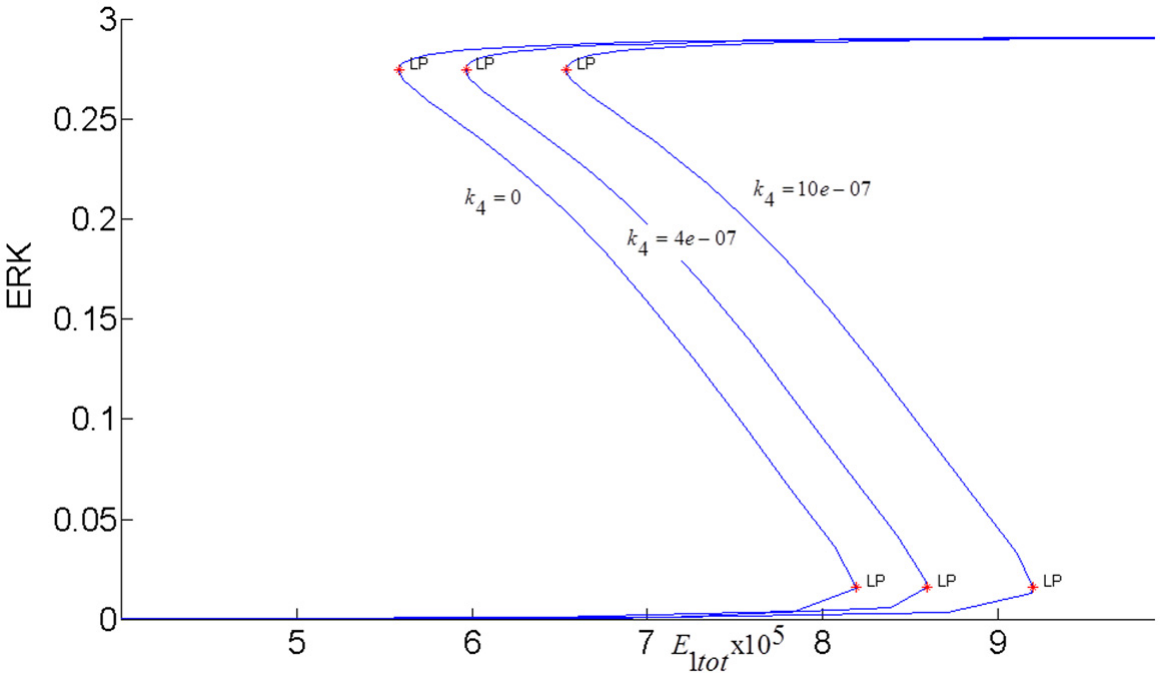
Steady-state ERK response curves for different pAKT inhibitions. *E*1_*tot*_ *=* 9×10^−5^. *k*_3_ *= 0*.

Active AKT *(pAKT*) phosphorylates RAF at serine residue *Ser*^259^ and inhibits the activation of the RAF-MEK-ERK signaling pathway [14]. Fig 9 shows that inhibition by pAKT shifts the ERK response curves to the right; therefore, stimulus *E*1_*tot*_ has to increase to sustain the bistable response and keep ERK activity. In case stimulus *E*1_*tot*_ is limited, ERK stays at its inactive state.

**Fig 10:**
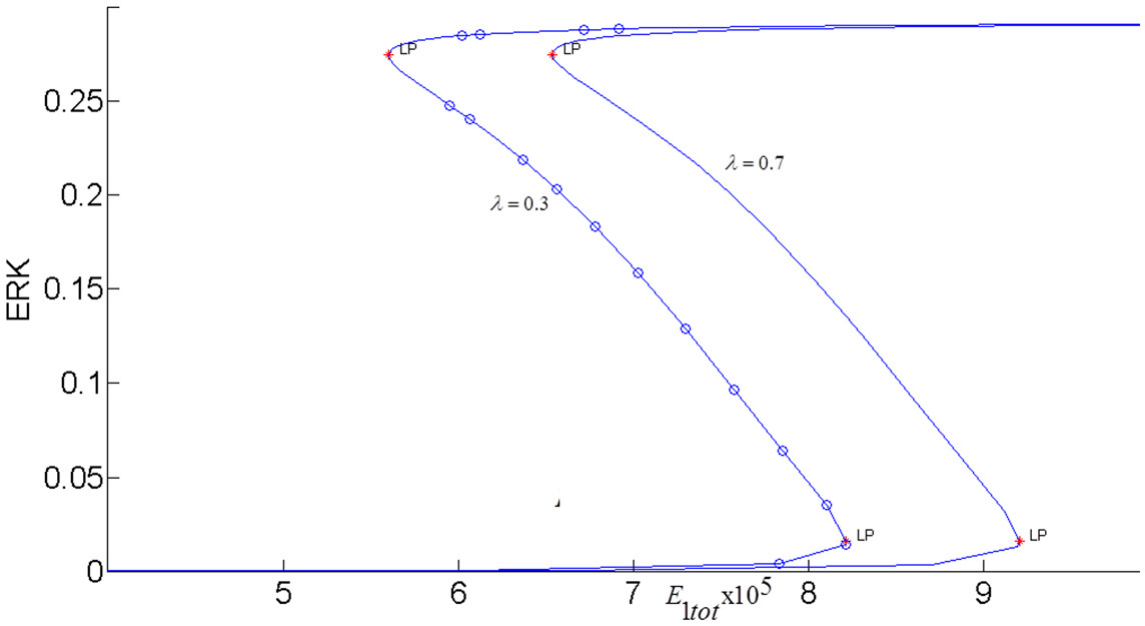
Steady-state ERK response curves when for different insulin levels. *k*_4_ *=* 1×10^−6^.

The effect of pAKT inhibition depends on the level of insulin as shown in Fig 10.

Lower insulin levels result in lower pAKT and less inhibition of ERK. Therefore, less E1 stimulus is needed to maintain the same ERK activity. It was also shown in [20] that pAKT suppresses RAF activity in a concentration dependent way with low doses of insulin growth factor hardly triggering the cross-talk.

#### Loss of PTEN disrupts bistability of both AKT and ERK signaling which induces proliferation of cancer cells

**Fig 11:**
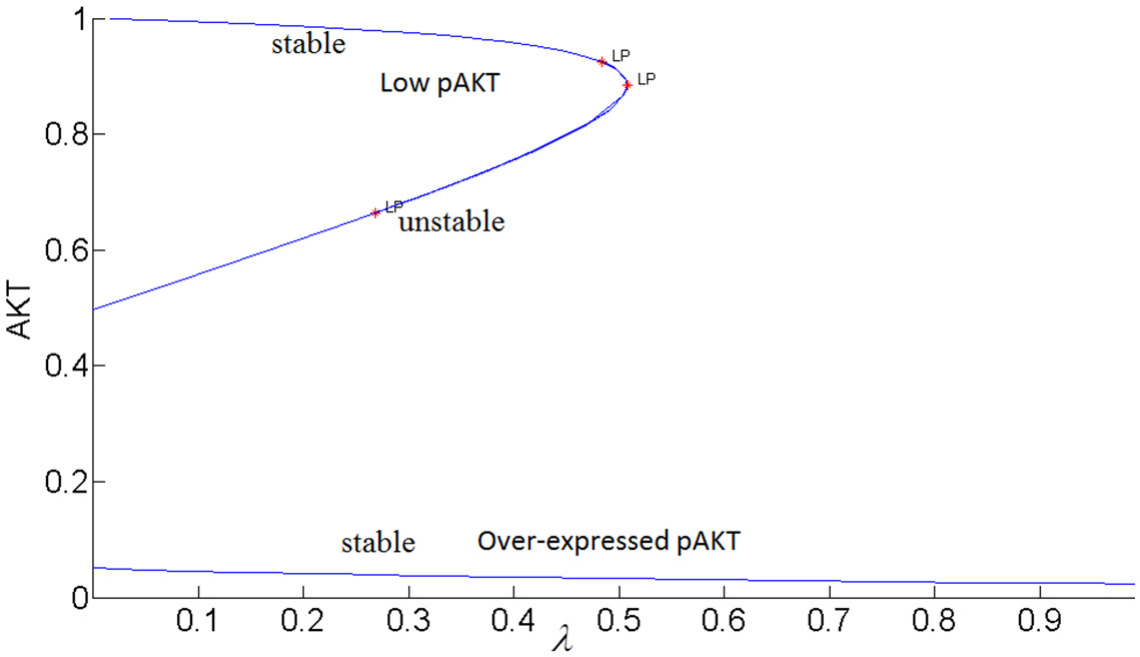
AKT response to insulin when *β* = 2. *k*_4_ = 1×10^−5^.

**Fig 12:**
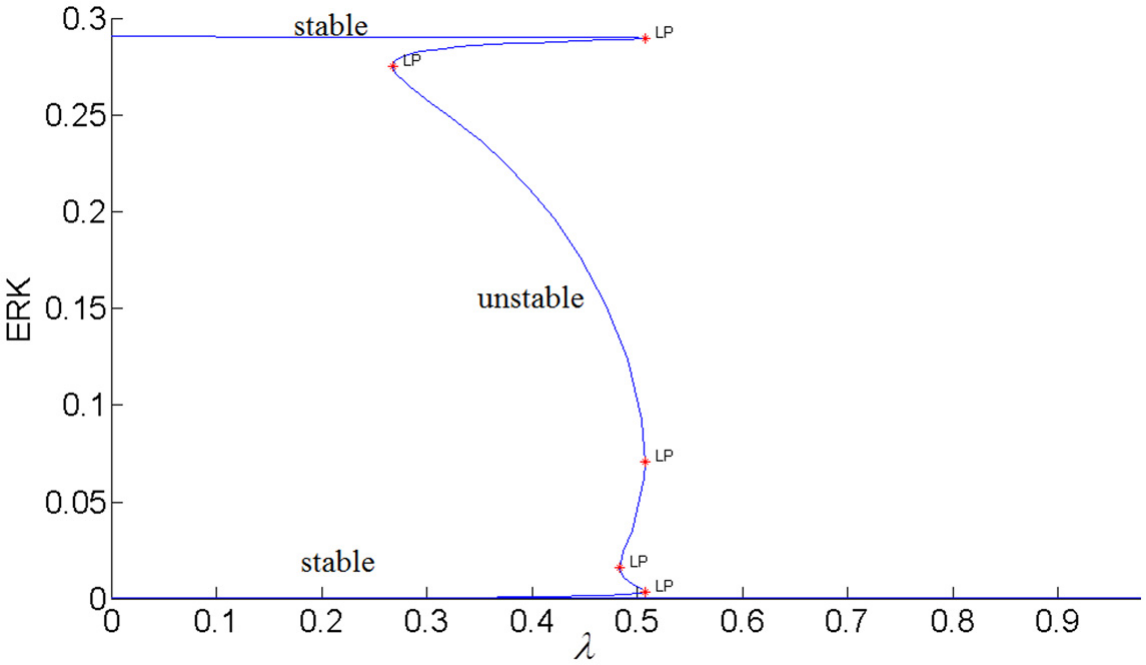
ERK responses to insulin when *β* = 2. *k*_4_ = 1×10^−5^.

The lipid protein phosphatase PTEN dephosphorylates PIP3 and negatively regulates AKT activation. PTEN protein suppresses tumor [51]. Mutations in PTEN gene result in various cancers. In order to propose an explanation for these observations based on our model predictions, we first simulated the loss of PTEN by increasing the feedback strength *β* (see Fig 2). It is shown in Fig 11 that two-way toggle switch is lost. At high insulin levels, pAKT is persistently overexpressed (i.e. AKT resides at its lower steady-state branch) and it cannot be decreased since the switch requires negative insulin which is not possible. Activated pAKT inhibits RAF, and ERK stays inactive at its low state as shown in Fig 12. Inhibition of RAF and ERK is followed by p53 and *p*21^*chip*1^ inactivation which shifts the cell cycle from growth arrest to proliferation as observed in androgen independent prostate cancer cells [22]. PTEN is the only insulin receptor phosphatase effect modeled in this work because of its significant biological functions cited above. If needed, the effects of other phosphatases can modeled and parameterized similarly to assess their significance.

*Closed-loop responses*: *k*_4_≠0 *and k*_3_≠0.

**Fig 13:**
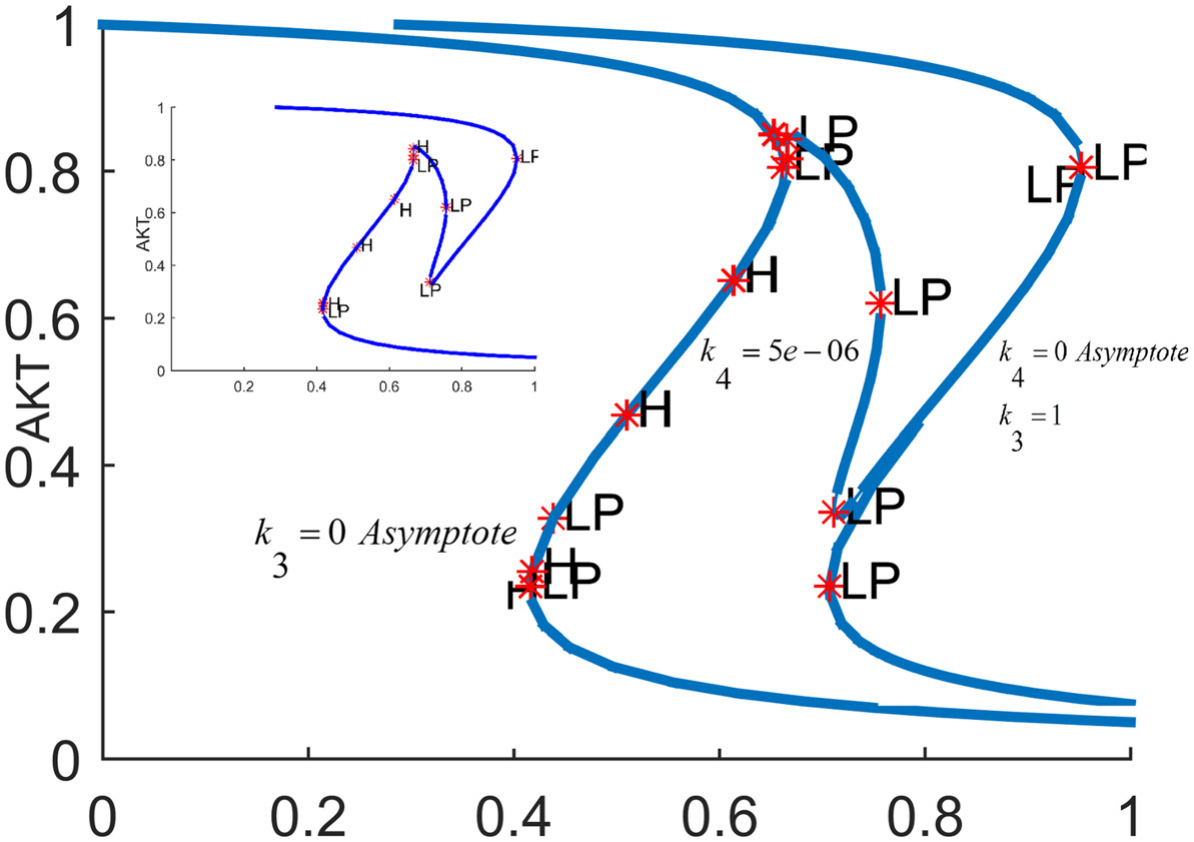
AKT response when the positive feedback loop *FB*(*k*_3_,*k*_4_) is closed.*E*_1*tot*_ *=* 9×10^−5^. Inset Fig is the response plotted without the asymptotes for clarity.

**Fig 14:**
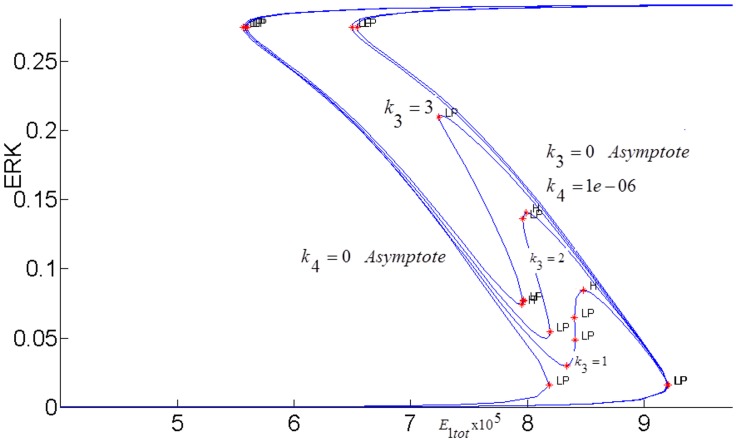
ERK responses to *E*1_*tot*_ modified by the interpathway positive feedback loop *FB*(*k*_3_,*k*_4_) *λ* = 0.8.

In order to fully assess the effect of the the positive feedback loop, both inhibitions in the loop must be simultaneously considered by setting both *k*_3_ and *k*_4_ to nonzero and closing the loop (see Fig 7). The closed-loop AKT response curves for different feedback strengths fall in between two open-loop asymptotes as shown in Fig 13. When there is no inhibition of pAKT by ERK, the response constitutes the open-loop non-inhibited “*k*_3_ = 0 *Asymptote*”. When there is no inhibition of ERK by pAKT, the response corresponds to the other open-loop maximally inhibited “*k*_4_ = 0 *Asymptote*”. The closed-loop pAKT activity lies between these asymptotes. It is bistable since the middle branch is unstable and is joined with stable lower and upper branches. The positive feedback has a similar effect on the response of EKT to its stimulus *E*1_*tot*_ as shown in Fig 14. Feedback responses are all bistable and lie between the maximally inhibited “*k*_3_ = 0 *Asymptote*” and the non-inhibited “*k*_4_ = 0 *Asymptote”*.

#### pAKT-ERK positive feedback loop regulates the balance between cell-arrest and proliferation

**Fig 15:**
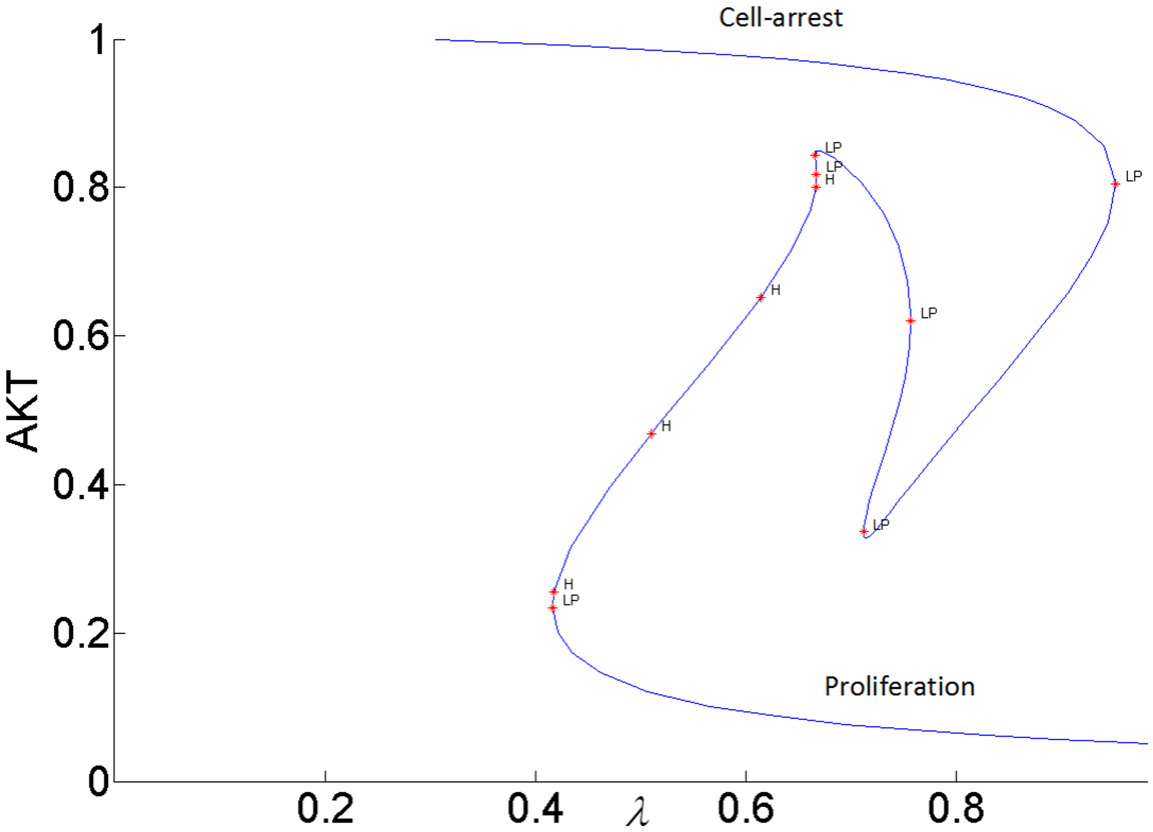
AKT response to insulin when *k*_3_ = 1 (higher inhibition of pIRS by ERK).

**Fig 16:**
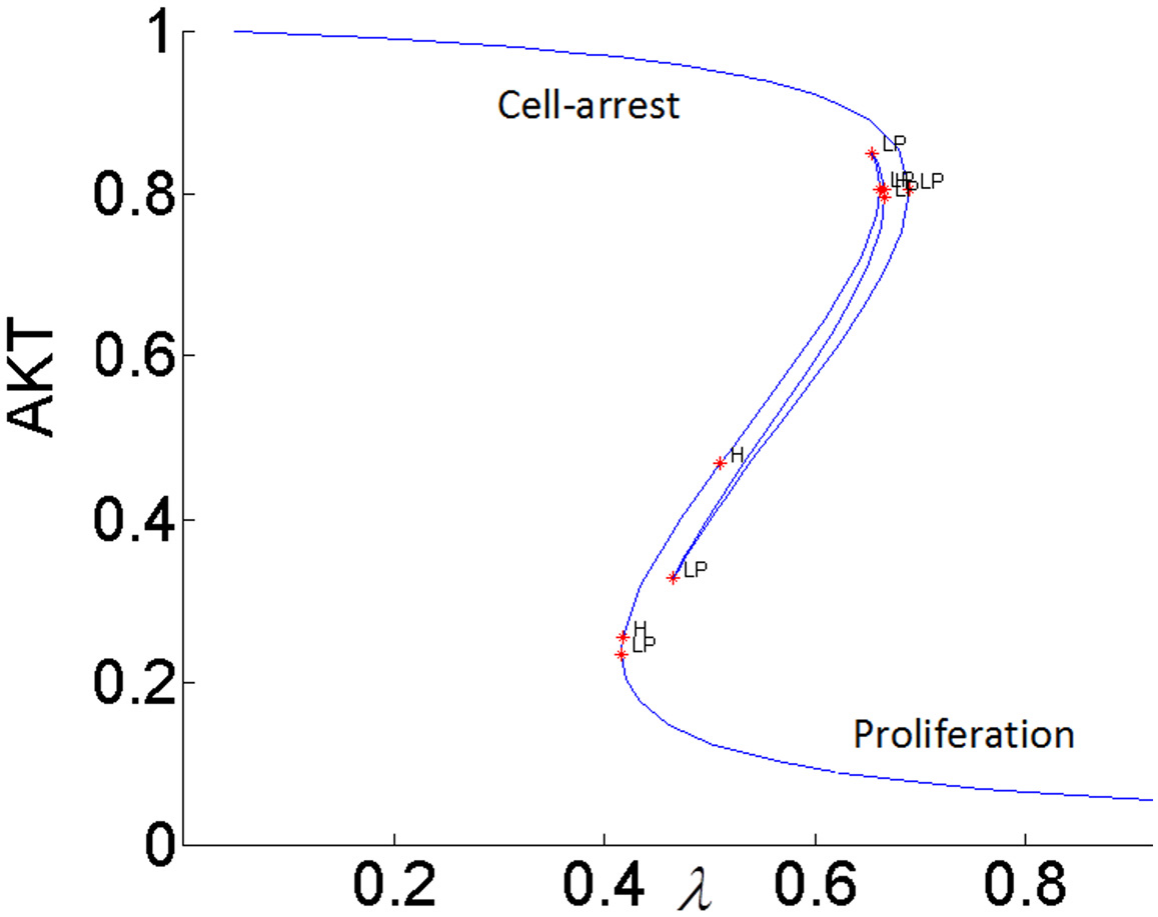
AKT response to insulin when *k*_3_ = 0.1 (lower inhibition of pIRS by ERK).

It has been proposed that highly active ERK promotes cell-cycle arrest by the induction of the cell-cycle inhibitors such as *p*21^*chip*1^, and low to moderate ERK activity stimulates proliferation and DNA synthesis through expression of cyclin D. [49,52–54]. Therefore, ERK’s signaling strength determines the type of cellular response by establishing different types of gene expression [52]. Here we propose that the switch between proliferation and cell-cycle arrest and the specificity of the cellular response are regulated by the feedback shown in Fig 7. In this positive feedback loop, pAKT inhibits cell-arrest and promotes proliferation by down regulating ERK. At the same time, ERK inhibits pAKT and reverts the cell-cycle from proliferation to arrest. Such a switching response is made possible due to the bistability established by the positive feedback. As shown in Figs 15 and 16, the lower steady-state branches of AKT (i.e. higher pAKT) and ERK correspond to cell proliferation, and higher steady-state branches of AKT and ERK represent cell-arrest. The cellular response can switch between these two stable states depending on the insulin level.

#### pAKT-ERK positive feedback loop together with ERK-RAS negative feedback loop provide flexibility to modulate ERK signal duration and magnitude

**Fig 17:**
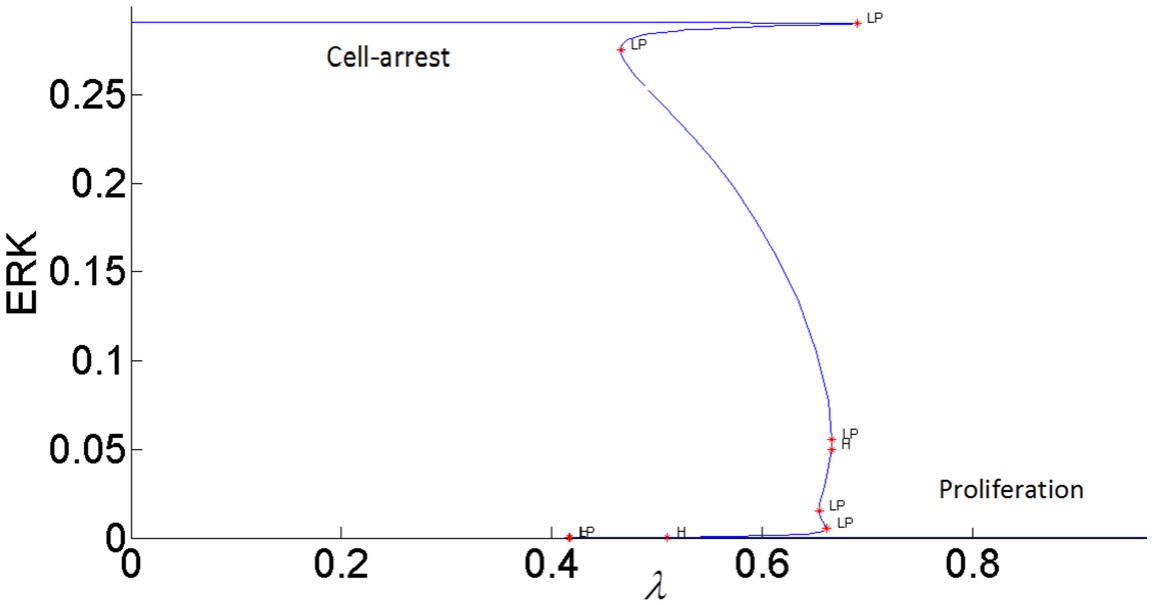
ERK response to insulin when *k*_3_ = 0.1 (lower inhibition of pIRS by ERK).

**Fig 18:**
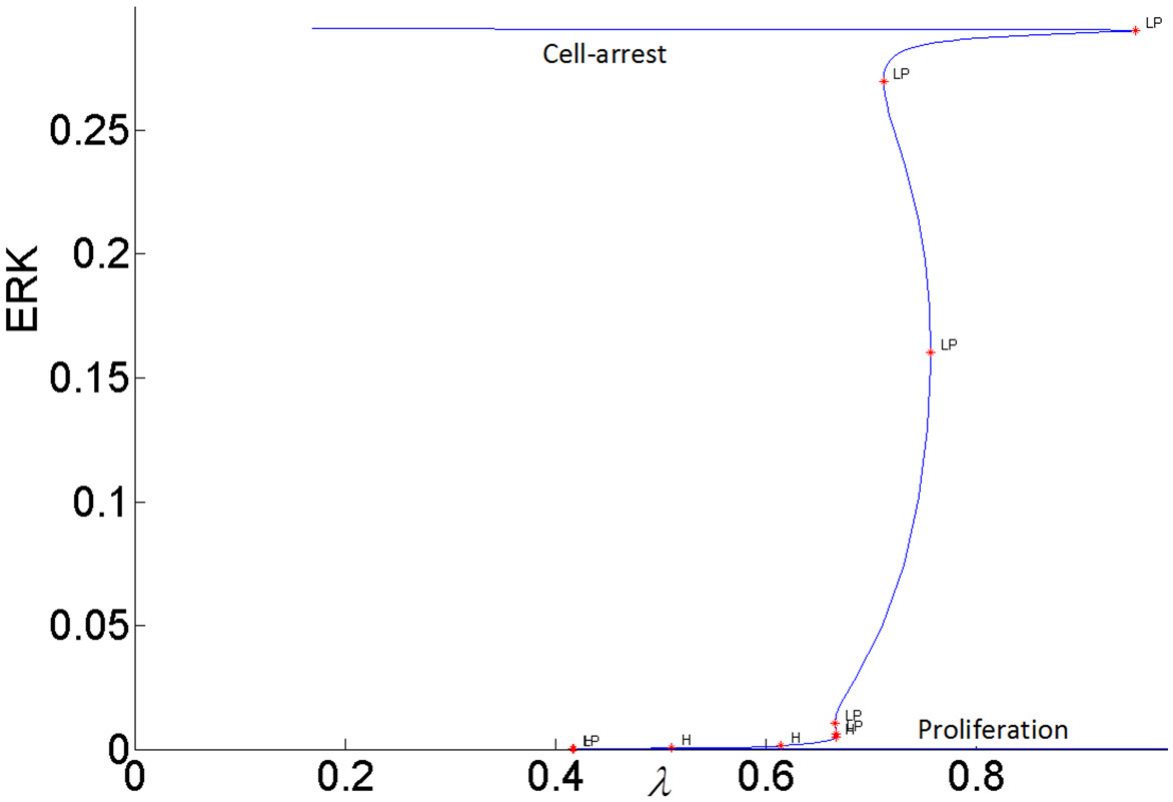
ERK response to insulin when *k*_3_ = 1 (higher inhibition of pIRS by ERK).

**Fig 19:**
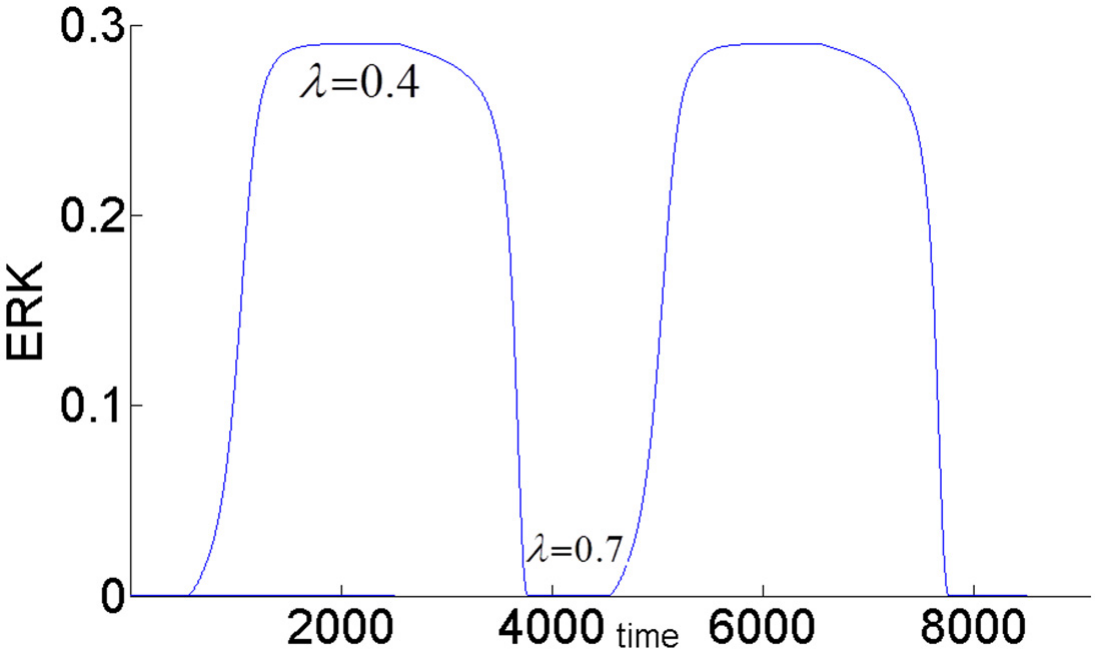
Dynamic response of ERK to a change in insulin showing the switching behavior. AKT switches similarly. *E*1_*tot*_ *=* 9×10^−5^. *k*_3_ = 0.1. For *k*_3_ = 1, AKT and ERK show sustained non-switching responses (both ERK and AKT rest at their high values) which are not shown in the Fig.

The feedback strength *k*_3_(or the level of ERK’s inhibition of pAKT) determines the duration of ERK’s activation. ERK signaling lasts longer for higher values of *k*_3_ i.e. ERK inhibits pAKT more, and this in turn helps to alleviate its inhibition by pAKT. In other words, it takes larger increase in insulin for cell-arrest to switch to proliferation. Comparing the switching (or turning) points of the bistable AKT curves in Figs 15 and 16, we see that insulin has to increase from 0.42 to 0.95 for *k*_3_ = 1 and from 0.42 to 0.70 for *k*_3_ = 0.1 for cell-arrest to switch to proliferation Similarly, for the opposite switch from proliferation to arrest, insulin has to decrease from 0.95 to 0.40 for *k*_3_ = 1 and from 0.70 to 0.40 for *k*_3_ = 0.1. Figs 17 and 18 show the similar switching behavior for ERK. Dynamic simulations confirm this behavior in Fig 19. Both AKT and ERK are able to switch when *k*_3_ = 0.1. For *k*_3_ = 1, AKT and ERK show sustained non-switching responses (both ERK and AKT rest at their high values) which is not shown in the figure.

**Fig 20:**
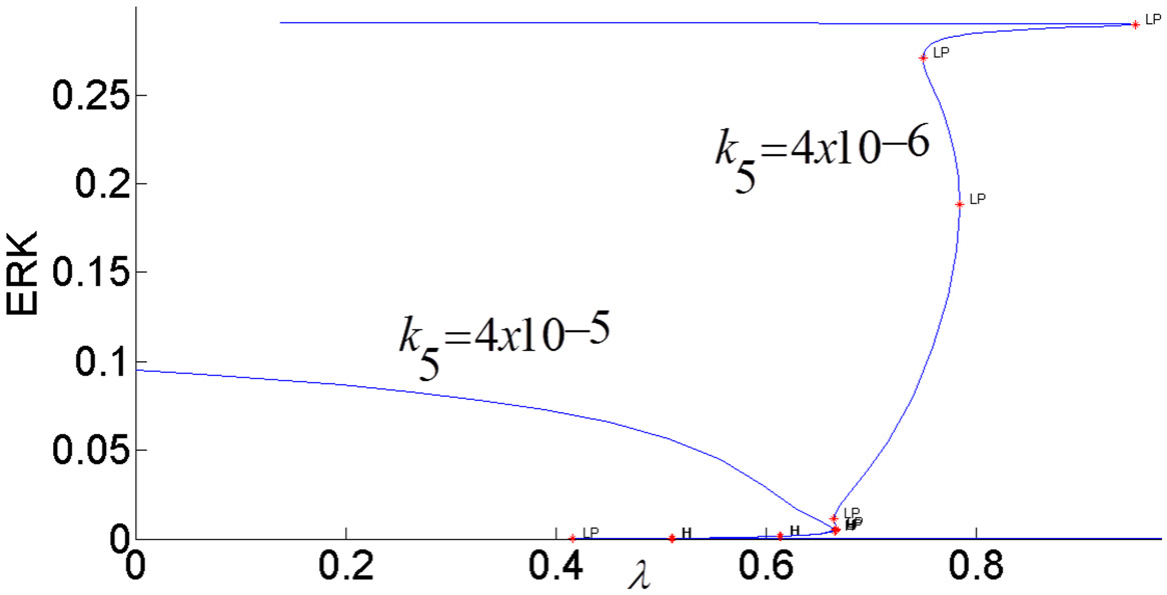
Effect of internal ERK-RAS negative feedback *k*_5_ on bistability.

While the feedback gain *k*_3_ (or pAKT inhibition by ERK) affects the duration of ERK signaling, the internal feedback gain *k*_5_ (or RAS inhibition by ERK) modulates the strength of ERK signaling as shown in Fig 20. When the negative feedback gain or RAS inhibition increases, the switching response gets suppressed as ERK is able to switch only between smaller magnitudes.

#### Positive pAKT-ERK feedback loop protects pAKT’s metabolic function against aberrant ERK activation

pAKT’s role in glucose transport can be adversely affected if it’s activation is seriously inhibited by ERK. The positive feedback loop can alleviate this effect if inhibitory action of pAKT counteracts ERK’s inhibition. If pAKT’s inhibition of ERK is disrupted (i.e. *k*_4_ = 0), the positive feedback loop is opened and pAKT becomes maximally inhibited by ERK and requires higher levels of insulin to perform its biological function. This seen in Fig 13 where the operating insulin level is 0.4–0.95 when positive feedback is active and 0.7–0.95 when it is disrupted.

**Fig 21:**
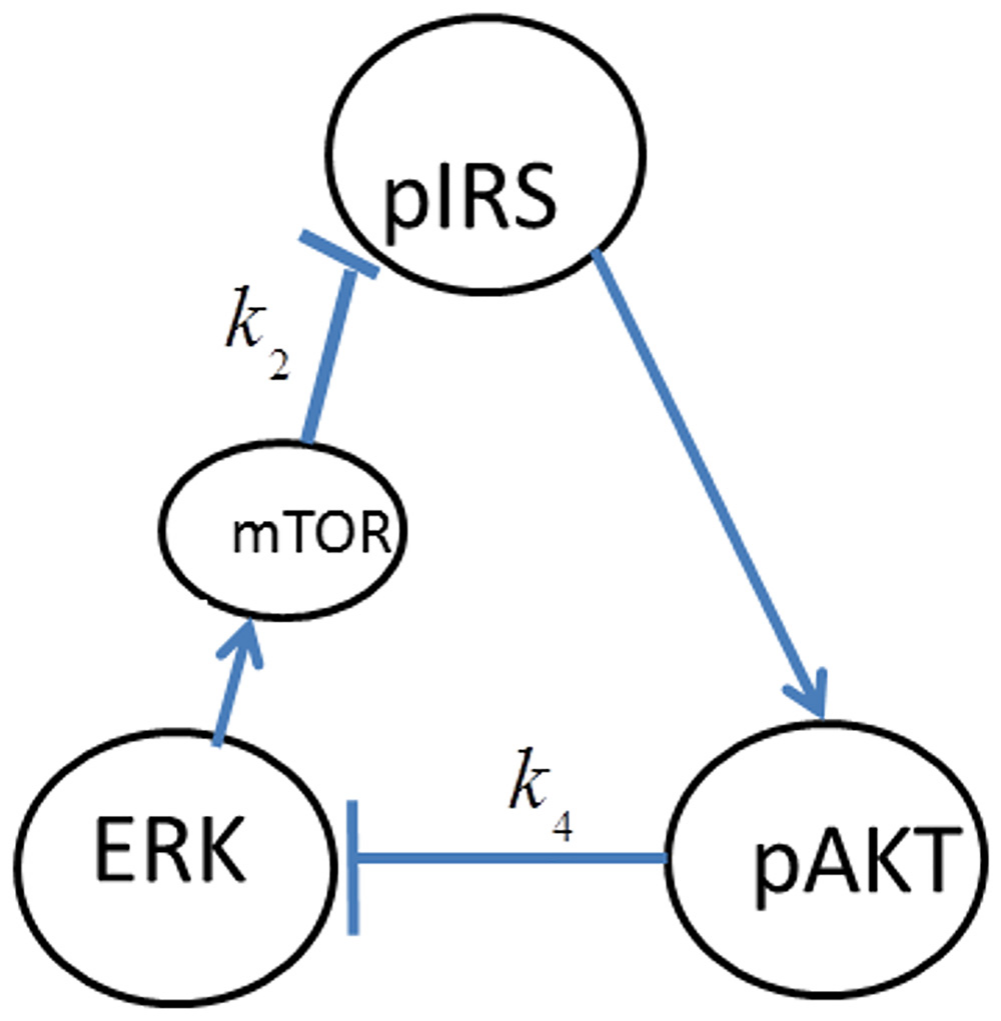
The positive feedback loop *FB*(*k*_2_,*k*_4_).

The second cross-talk positive feedback loop is *pIRS1→pAKT→pERK→mTOR→pIRS1* which is represented by *FB*(*k*_2_,*k*_4_) and shown in Fig 21. mTOR plays a major role in this cross-talk. mTOR regulates cell growth by sensing nutrients and mitogenic signals and it stimulates protein synthesis. Deregulation of mTOR signaling is involved in the development of cancer, type-2 diabetes and obesity [24].

#### ERK-mTOR-AKT positive feedback loop controls the insulin sensitivity

**Fig 22:**
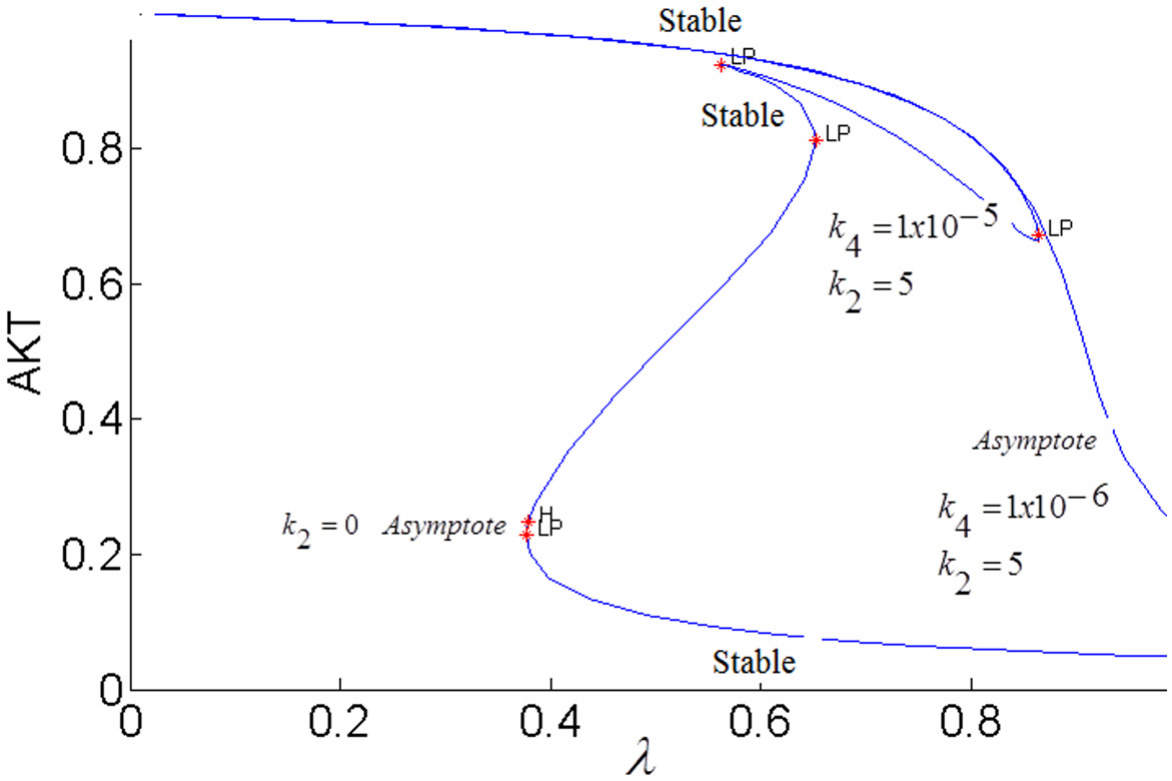
AKT response of the positive feedback loop *FB*(*k*_2_,*k*_4_). *E*_1tot_ *=* 9×10^−5^.

Activated ERK phosphorylates TSC2 which increases the activation of mTOR. mTOR activates S6K which inhibits IRS1 and pAKT [24, 26, 28]. Fig 22 shows that, when ERK’s inhibition dominates pAKT’s inhibition (*k*_2_ = 5,*k*_4_ = 10^−6^), insulin sensitivity is lost as the steady-state curve no longer exhibits the *S* shape bistable response curve. Irreversible loss of insulin sensitivity can lead to type-2 diabetes. When pAKT’s inhibition of ERK is increased (*k*_2_ = 5,*k*_4_ = 10^−5^), bistability and insulin sensitivity are restored. When the two cross-talks (or inhibitions) cooperate in the positive feedback loop, pAKT activity can switch between its lower and upper stable branches at the insulin levels of 0.37 and 0.85 corresponding to the turning points of the feedback response curve and perform its biological function. Otherwise, over-expressed ERK results in hyper-active mTOR and insulin insensitivity.

**Fig 23:**
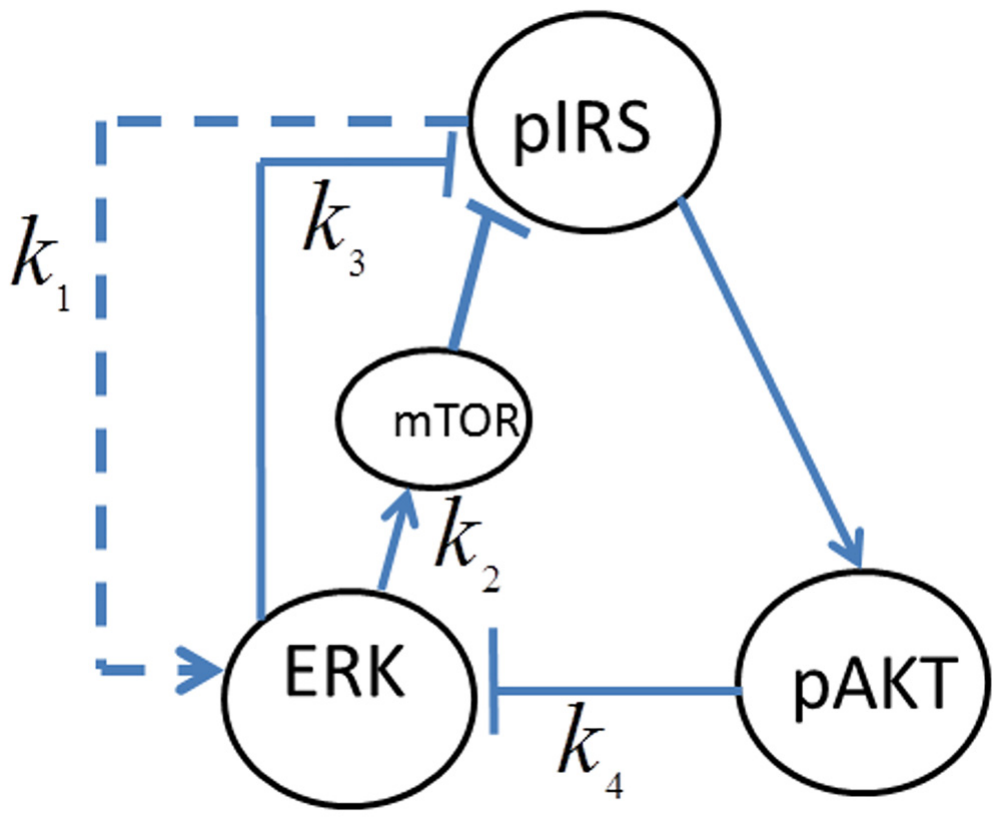
The negative feedback loops *FB*(*k*_1_,*k*_3_) and *FB*(*k*_1_,*k*_2_) functioning together with the positive loops.

In addition to the two positive feedback loops, ERK activation by pIRS introduces two negative feedback loops as shown in Fig 23. These inter-pathway loops are *pIRS→ERK→ pIRS and pIRS→ERK→mTOR→pIRS* negative feedback loops represented by *FB*(*k*_1_,*k*_3_) and *FB*(*k*_1_,*k*_2_), respectively (refer to Fig 23 for the notation).

#### Activation of the Grb2-SOS complex by insulin reduces the strength of the core positive feedback and increases the inhibition of pAKT

**Fig 24:**
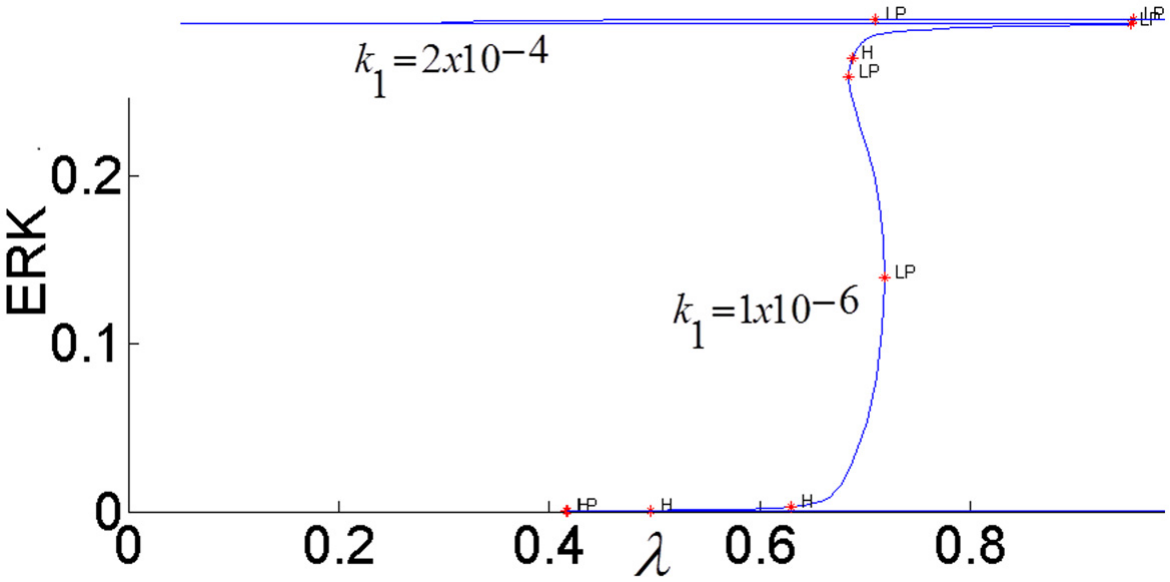
EKT responses for different strengths (*k*_1_) of ERK activation by pIRS1.

**Fig 25:**
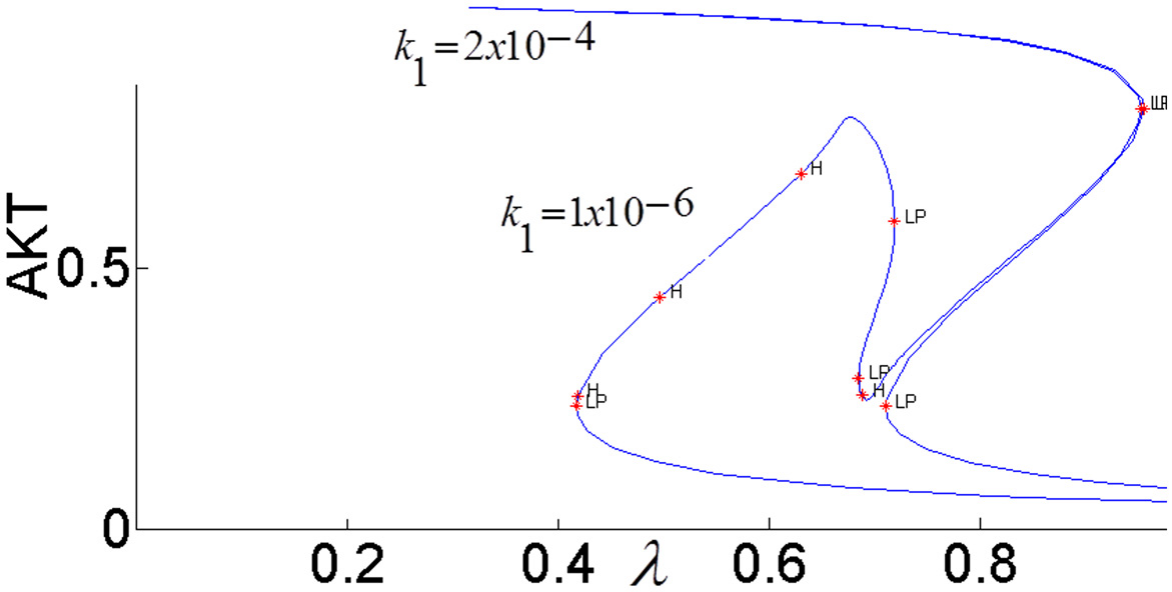
AKT responses for different strengths (*k*_1_) of ERK activation by pIRS1.

In general, a negative feedback loop can adversely affect the useful functions of a positive feedback loop [55]. In the case of AKT-MAPK cross-talk, upon insulin’s binding to its receptor IR, the tyrosine phosphorylation of its substrate IRS1 is promoted. Tyrosine phosphorylated IRS1 (pIRS1) stimulates the MAPK signaling cascade by catalyzing Shc which interacts with the Grb2-SOS complex. This is followed by the activation of ERK. If this activation is strong enough to counteract pAKT’s inhibition of ERK, pIRS and pAKT become maximally inhibited. As shown in Fig 24, when the Grb2-SOS complex is over-active (*k*_1_ *=* 2×10^−4^), ERK signal saturates at its maximum value. This results in increased inhibition of pIRS by ERK, and pAKT response asymptotically approaches the maximally inhibited response as shown in Fig 25. Reducing the activation of the Grb2-SOS complex (*k*_1_ *=* 1×10^−6^) recovers the desired bistable response.

#### ERK activation caused by mTOR inhibition (e.g. upon ramapycin treatment) can be reduced if Gab1’s association with PI3K is inhibited

**Fig 26:**
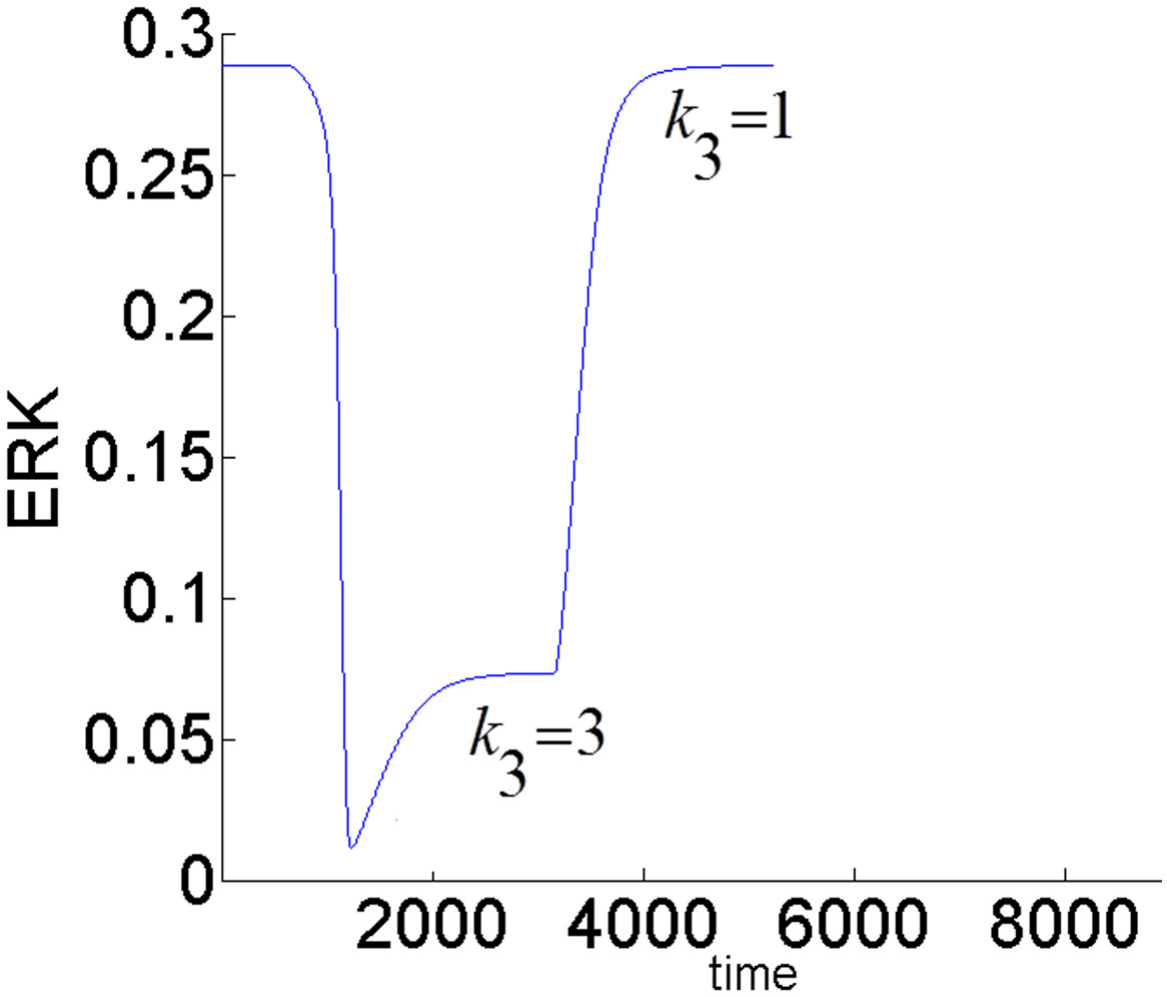
Inhibition introduced in the ERK-Gab1-PI3K pathway (e.g. increasing *k*_3_) alleviates the activation of ERK due to mTOR inhibition (after ramapycin treatment).

The aberrant activity of mammalian target of ramapycin (mTOR) is connected with various cancers. In [28] it has been shown that mTOR inhibitor ramapycin increases the activation of MAPK as measured by phosphorylated ERK in both normal cells and human cancer cell lines. Inhibition of mTOR by ramapycin activates both pIRS1 and AKT and it stimulates the MAPK signaling cascade by activating the Grb2-SOS complex (see Fig 1). This is followed by the activation of ERK. ERK-Gab1-PI3K inhibition (*k*_3_) forms a negative feedback loop with pIRS-Grb2-SOS-ERK activation (*k*_1_) denoted as *FB*(*k*_1_,*k*_3_) in Fig 23. Thus, any increased inhibition introduced in the ERK-Gab1-PI3K pathway (e.g. PI3K inhibitor) will alleviate the activation of ERK due to mTOR inhibition. Such a scenario is created by the simulating the model and the results are shown in Fig 26. Initially, ramapycin is used to inhibit mTOR. This is achieved by setting ε = 0 and *k*_2_ = 0 in the model. pIRS is upregulated and both AKT and ERK are activated. As shown in Fig 26, ERK activity stays at its maximum value of 0.289 initially. At time = 500, inhibition through ERK-Gab1-PI3K pathway is increased by increasing the feedback gain (*k*_3_ = 3). As a result, ERK activation is reduced. Any subsequent release of the inhibition (*k*_3_ = 1) up regulates ERK. Examining the cross-talk identifies that both pathways (AKT and ERK) are overactive during mTOR activation, and using a combination of mTOR and MAPK inhibitors can provide additional benefit for clinical treatment of human cancer as suggested in [22,56, 57].

## Conclusions

In this work we have developed a new mathematical model to study the steady-state and dynamic characteristics of the major feedback loops that regulate the cross-talk between insulin-AKT and MAPK/ERK signaling pathways. Significant amount of biological knowledge from the literature was used and earlier models for the AKT [34, 40] and MAPK [32, 41] pathways were combined by modeling the major cross-talk interactions between these two signaling networks. During the process of modeling, negative and positive feedback loops were identified and their effects on the responses of the individual pathways were analyzed by simulations.

Various biological functions of the AKT and MAPK pathways are performed and sustained through an intricate coordination among different negative and positive feedback loops. Any mutations and alterations in the strength of the feedback signals involved in the cross-talk can easily lead to undesired multiple over-activations of the individual pathways leading to disease states. Under normal conditions, the internal feedbacks within the AKT pathway function in tandem to provide a bistable switch-like response which is necessary for the glucose transport. MAPK cascade can have bistable response as well due to the phosphorylation–dephosphorylation cycle. The negative feedback inhibition within the MAPK cascade affects the duration, strength and sensitivity of ERK’s response to external stimulus.

Close analysis of the cross-talk reveals two negative and two positive inter-pathway feedback loops operating in tandem with one negative feedback loop within the MAPK pathway and two feedback loops (one negative and one positive) within the AKT pathway. These embedded feedback loops determine the desired response characteristics such as sensitivity and bistability.

For the AKT-MAPK system under study, positive inter-pathway loops exist because ERK inhibits AKT and AKT inhibits ERK within the same feedback loop. There are two such cross-talk loops since ERK inhibits AKT by two different mechanisms. One is through activating mTOR and the other is by down-regulating the association of Gab1 with PI3K. The model shows that if ERK inhibits AKT via mTOR excessively, bistable response of AKT to insulin can be lost. However, positive feedback restores bistability since pAKT inhibits ERK, and this in turn helps to alleviate its inhibition by ERK. When ERK inhibits AKT through Gab1, bistability is maintained but AKT requires higher levels of insulin to perform its biological function. In this case, positive feedback helps to increase the sensitivity of AKT to insulin. Thanks to these two positive feedback loops, both AKT and ERK can exhibit switch-like responses to their growth factors under normal conditions.

The negative inter-pathway feedback reduces the strength of the two core positive inter-pathway feedbacks. Tyrosine phosphorylated IRS1 (pIRS1) stimulates the MAPK signaling cascade and activates ERK. If this activation is strong enough to compensate for pAKT’s inhibition of ERK, pAKT becomes maximally inhibited. Bistability can be lost leading to insulin insensitivity and type-2 diabetes.

We cannot compare our simulation results vis-a-vis with other modeling studies since, to the best of our knowledge, such an analytical model for the cross-talk does not exist in the literature. But, most importantly, different simulation scenarios show that for the chosen parameter ranges the model can predict the experimental or clinical observations of physiological behaviors of normal and diseased states reported in the literature. The model includes the most important interactions cited in the literature and the effects of certain intermediate interactions are either ignored or lumped in order to facilitate the computations and the subsequent analysis. Similar simplifications have been made for the individual AKT and MAPK models in the literature for the same reasoning. As the simulations results show, the model can explain the important trends reported in the literature by proposing new feedback mechanisms. A more detailed analysis and model validation can be easily performed by including other interactions, if needed. The structure of the model is open to such future enhancements.

Using the developed model, we produce several new hypotheses that link some of the important literature findings to the working mechanisms of the underlying feedback loops. These hypotheses are tested in silico, and results should hopefully pave the way for future experimental design and validation.

Parameter values for the AKT and MAPK models were taken from the literature. Since reliable, true values of the new cross-talk interaction parameters are not available, these parameters were changed within certain ranges and their effects were assessed. In order to be able to estimate the cross-talk interaction parameters used in this work and further validate the model, reliable measurements will be helpful in the future.

## Supporting Information

S1 Text Derivation of the model. (DOCX)
