# Supplementary material for: Dynamic Modeling and Analysis of the Cross-Talk between Insulin/AKT and MAPK/ERK Signaling Pathways
Source: PLoS One. 2016 Mar 1;11(3):e0149684. doi: 10.1371/journal.pone.0149684 (PMC4773096; doi:10.1371/journal.pone.0149684)
Supplement: S1 Text — (DOCX) [file pone.0149684.s001.docx]

SUPPORTING INFORMATION

**S1 Text**

First we consider the Insulin/AKT pathway without any cross-talk. Modeling equations are based on the phosphorylation and dephosphorylation cycle (PdPC) of AKT ⮀ pAKT :

where E_1_ and E_2_ are the enzymes. Assuming Michaelis-Menten kinetics, the following dynamical model is derived [34]:

| $\frac{d\left[AKT\right]}{dt}= -a_{1}\left[AKT\right]\left[E_{1}\right]+ d_{1}\left[AKT:E_{1}\right]+ k_{2}\left[pAKT:E_{2}\right]$ | (1) |
| --- | --- |
| $\frac{d\left[AKT:E_{1}\right]}{dt}=a_{1}\left[AKT\right]\left[E_{1}\right]- \left(d_{1}+k_{1}\right)\left[AKT:E_{1}\right]$ | (2) |
| $\frac{d\left[pAKT\right]}{dt}= -a_{2}\left[pAKT\right]\left[E_{2}\right]+ d_{2}\left[pAKT:E_{2}\right]+ k_{1}\left[AKT:E_{1}\right]$ | (3) |
| $\frac{d\left[pAKT:E_{2}\right]}{dt}=a_{2}\left[pAKT\right]\left[E_{2}\right]- \left(d_{2}+ k_{2}\right)\left[pAKT:E_{2}\right]$ | (4) |
| $\frac{d\left[pIRS1\right]}{dt}= \gamma+\left(Ф-\text{ε} \Psi\right)\left[pAKT\right]- \delta[pIRS1]$ | (5) |
| with |  |
| $X_{T}=\left[AKT\right]+ [pAKT]$ (concentration of total AKT) | (6) |
| $E_{1T}=\left[E_{1}\right]+ \left[AKT:E_{1}\right]$ (concentration of total $E_{1}$) | (7) |
| $E_{2T}=\left[E_{2}\right]+ \left[pAKT:E_{2}\right]$ (concentration of total $E_{2}$) | (8) |
| $E_{1T}=\beta\left[pIRS1\right]$ | (9) |

Variable $\gamma$ denotes the insulin level and $\psi$ is the nutrient level. The critical parameters $\beta,\Phi\text{and} \varepsilon$ represent the strengths of the feedback loops as shown in Figure 3. The rest of the model parameters are constant physical parameters representing the reaction rates and the decay rate of pIRS1.

Define the following variables:

$$x_{1}=\left[AKT\right]/X_{T}; x_{2}=\left[AKT:E_{1}\right]/X_{T}; x_{3}=\left[pAKT\right]/X_{T}; x_{4}=\left[pAKT:E_{2}\right]/X_{T}; x_{5}=\left[pIRS\right]/X_{T}$$

where $X_{T}=\left[AKT\right]+\left[pAKT\right]$

Then Equations (1) - (5) can be expressed in terms of the above variables:

$\frac{dx_{1}}{dt}=\frac{-(d_{1}+k_{1})}{K_{1}}x_{1}(\beta x_{5}-x_{2})+d_{1}x_{2}+k_{2}x_{4}$ (10)

$\frac{dx_{2}}{dt}=\frac{(d_{1}+k_{1})}{K_{1}}x_{1}(\beta x_{5}-x_{2})-(d_{1}+k_{1})x_{2}$ (11)

$\frac{dx_{3}}{dt}=\frac{-(d_{2}+k_{2})}{K_{2}}x_{3}(\tilde{E}_{2T}-x_{4})+d_{2}x_{4}+k_{1}x_{2}$ (12)

$\frac{dx_{4}}{dt}=\frac{(d_{2}+k_{2})}{K_{2}}x_{3}(\tilde{E}_{2T}-x_{4})-(d_{2}+k_{2})x_{4}$ (13)

$\frac{dx_{5}}{dt}=(\frac{\delta}{\beta})(\frac{k_{2}}{k_{1}})\tilde{E}_{2T}\lambda+(\Phi-\varepsilon\Psi)x_{3}-\delta(d_{1}+k_{1})x_{5}$ (14)

where $\tilde{E}_{2T}=E_{2T}/X_{T}$ and $\lambda= \frac{\gamma\beta k_{1}}{\delta k_{2}E_{2T}}$.

Assuming that pseudo steady-state holds for the AKT and pAKT complexes, (11) and (13) are set equal to zero and solved for:

$x_{2}=\frac{\beta x_{5}x_{1}}{K_{1}+x_{1}}$ and (15)

Since $X_{T}=\left[AKT\right]+\left[pAKT\right]$, it also follows that $x_{1}+x_{3}=1$ or

$x_{3}=1-x_{1}$ (16)

Substituting (15) and (16) into (10)-(14) one obtains:

(17)

$\frac{dx_{5}}{dt}=(\frac{\delta}{\beta})(\frac{k_{2}}{k_{1}})\tilde{E}_{2T}\lambda+(\Phi-\varepsilon\Psi)(1-x_{1})-\delta(d_{1}+k_{1})x_{5}$ (18)

Next the cross-talk terms due to ERK’s inhibition are included in (17) and (18):

(19)

The first new term is :$-\Psi\bar{k}_{2}\left[pERK\right](1-x_{1}).$This negative feedback term denotes the inhibition of IRS1 by mTOR/S6K. As a mechanism we have assumed that pAKT or $(1-x_{1})$ and pERK jointly phosphorylate the TSC1/2 complex and activate mTOR. Therefore the strength of the negative feedback signal depends on pERK and pAKT levels. The second interaction from MAPK pathway $-\bar{k}_{3}\left[ERK\right]$is the direct inhibition of pIRS by ERK which down-regulates Gab1’s association with PI3K

In terms of the original variables, the final model is given by:

(20)

(21)

Next we consider the MAPK pathway without any cross-talk. Modeling equations are based on the following phosphorylation and dephosphorylation reactions [41]:

$$KKK+E1$$

$$KKK^{*}+E2$$

$$KK+KKK^{*}$$

$$KK-P+KKP'ase$$

$$KK-P+KKK^{*}$$

$$KK-PP+KKP'ase$$

$$K+KK-PP$$

$$K-P+KP'ase$$

$$K-P+KK-PP$$

$$K-PP+KP'ase$$

The dynamic model model is given in [32]. Here we include the cross-talk interactions and the internal feedback to that model:

(22)

(23)

$\frac{d\left[KKK^{*}.E2\right]}{dt}=a_{2}\left[KKK^{*}\right]\left[E2\right]-(d_{2}+k_{2})\left[KKK^{*}.E2\right]$ (24)

$\frac{d\left[KK.KKK_{*}\right]}{dt}=a_{3}\left[KK\right]\left[KKK^{*}\right]-(d_{3}+k_{3})\left[KK.KKK^{*}\right]$ (25)

(26)

(27)

$\frac{d\left[KK-P.KKK^{*}\right]}{dt}=a_{5}\left[KK-P.KKK^{*}\right]-(d_{5}+k_{5})\left[KK-P.KKK^{*}\right]$ (28)

(29)

(30)

$\frac{d\left[K..KK-PP\right]}{dt}=a_{7}\left[K\right]\left[KK-PP\right]-(d_{7}+k_{7})\left[K.KK-PP\right]$ (31)

(32)

(33)

$\frac{d\left[K-P.KK-PP\right]}{dt}=a_{9}\left[K-P\right]\left[KK-PP\right]-(d_{9}+k_{9})\left[K-P.KK-PP\right]$ (34)

(35)

(36)

Above 15 differential equations are solved together with the two differential equations (20) and (21).

The output of the MAPK cascade is $K-PP$ which is the phosphorylated *ERK*. The model includes three cross-talk terms and one internal feedback inhibition from ERK to RAS in equations (22) and (23):

$\bar{k}{}_{1}\left[pIRS1\right]:$insulin dependent activation of RAS.

$\bar{k}_{4}\left[1-AKT\right]$: inhibition of RAF by pAKT

$\bar{k}_{5}\left[KK-PP\right]$: inhibition of RAS by internal feedback from ERK.

The bars on the interaction parameters *k*’s are used to differentiate them from the other *k*’s which appear as the model parameters. In the sequel, the bars are dropped.

In the simulations the input stimulus to the MAPK cascade is $E1_{tot}$.

**Table A. Parameter Values**

| **AKT Parameters** | **Nominal**  **Values [33]** | **MAPK**  **Parameters** | **Nominal**  **Values [31]** | **MAPK Parameters** | **Nominal**  **Values [31]** |
| --- | --- | --- | --- | --- | --- |
|  | 0.0909 |  | 3.3723e+002 |  | 1.4607e+002 |
|  | 0.0909 |  | 1.8410e+003 |  | 3.3844e+002 |
|  | 0.9091 |  | 1.2260e+003 |  | 4.2000e+002 |
|  | 0.9091 |  | 2.9603e+003 |  | 6.6820e+002 |
|  | 0.05 |  | 3.3837e+003 |  | 2.1465e+002 |
|  | 0.05 |  | 1.9568e+003 |  | 6.7970e+001 |
| $\beta$ | 1 |  | 2.2957e+002 |  | 4.3658e+001 |
| $\Phi$ | 1 |  | 2.9700e+002 |  | 3.1743e+001 |
| $\Psi$ | 0.559 |  | 3.3887e+002 |  | 6.5732e+001 |
|  | 1 |  | 9.7470e+002 |  | 1.7591e+002 |
| $\varepsilon$ | 0.01 |  | 2.6110e+002 | $MAPKKK_{tot}$ | 9.2235e-004 |
|  | 1 |  | 1.9847e+002 | $MAPKK_{tot}$ | 5.1288e+000 |
|  |  |  | 6.2317e+002 | $MAPK_{tot}$ | 8.1552e-001 |
|  |  |  | 1.6300e+002 | $E1_{tot}$ | 7.9433e-005 |
|  |  |  | 6.0531e+002 | $E2_{tot}$ | 3.2830e-004 |
|  |  |  | 4.8804e+001 | $MAPKKP'ase_{tot}$ | 2.1238e-004 |
|  |  |  | 6.9413e+002 | $MAPKP'ase_{tot}$ | 5.0345e-001 |
|  |  |  | 3.0109e+002 |  |  |
|  |  |  | 4.8535e+002 |  |  |
|  |  |  | 5.8745e+002 |  |  |

**Table B. Units**

| $MAPKKK_{tot}$ | $\mu M$ |
| --- | --- |
| $MAPKK_{tot}$ | $\mu M$ |
| $MAPK_{tot}$ | $\mu M$ |
| $E1_{tot}$ | $\mu M$ |
| $E2_{tot}$ | $\mu M$ |
| $MAPKKP'ase_{tot}$ | $\mu M$ |
| $MAPKP'ase_{tot}$ | $\mu M$ |
| $a_{i}$ | $(\mu M.\min)^{-1}$ |
| $d_{i}$ | $\min^{-1}$ |
| $k_{i}$ | $\min^{-1}$ |
|  | $\mu M$ |
|  | $\mu M$ |
| $\Phi$ | $\min^{-1}$ |
| $\Psi$ | $\mu M$ |
|  | $\min^{-1}$ |
| $\varepsilon$ | $(\mu M.\min)^{-1}$ |
